# High performance fake review detection using pretrained DeBERTa optimized with Monarch Butterfly paradigm

**DOI:** 10.1038/s41598-025-89453-8

**Published:** 2025-03-03

**Authors:** S. Geetha, E. Elakiya, R. Sujithra Kanmani, Manas Kamal Das

**Affiliations:** School of Computer Science and Engineering, Vellore Institute of Technology, Chennai, Tamilnadu 600127 India

**Keywords:** Neural network, Deep learning, Fake review detection, DeBERTa, Monarch Butterfly, Engineering, Mathematics and computing

## Abstract

In this era of internet, e-commerce has grown tremendously and the customers are increasingly relying on reviews for product information. As these reviews influence the purchasing ability of the future customer, it can give a positive or negative impact on the businesses. The effectiveness of online reviews is compromised by fake reviews that provide false information about the product. Fake reviews can not only impact the reputation of the businesses but also involve financial losses. Thus, detection of fake reviews is essential to solve the problem for maintaining the integrity of online reviews. Existing Machine learning models often struggle with deep contextual understanding. Scaling machine learning models while maintaining accuracy and efficiency becomes increasingly challenging as the volume of online reviews continues to grow. Hence, this research work introduces a novel MBO-DeBERTa, a deep neural network with Monarch Butterfly Optimizer. The proposed model improves the capacity to differentiate between overlapping characteristics of fake and authentic reviews. MBO-DeBERTa attained a classification accuracy of 98% for detecting the fake reviews. The proposed framework is tested on three different datasets such as Amazon, Fake Review and Deceptive Opinion Spam containing 21000,40000 and 1600 reviews respectively which are publicly available in Kaggle. The proposed model also detects adversarial attacks using the Fast Gradient Sign Method (FGSM) and thereby evaluating its resistance to such attacks and noise. The proposed model was also tested on the unseen data of Myntra and Amazon verified customer reviews and our model works efficiently for real world data. Thus the results show that the suggested model outperforms the current models showing increased accuracy, precision, recall, F1 score and reduced loss rate.

## Introduction

A review is a concise summary of feedback, opinion, or evaluation about a product, service or experience usually provided by a customer or user. In the current era of e-commerce and online buying the user generated content such as online reviews are the oils to the gears of recommendation system. This review has a direct proportionality to the growth of the business and ecommerce. It is beneficial to both the consumers and producers of the ecommerce platform. Fake reviews can have a significant impact on customer behaviour and ultimately damage the credibility of the platform because consumers are relying more and more on online platforms to make purchasing decisions. But due to the collaborative filtering the problem of fake reviews has spread widely. A fake review is one that has been created or altered artificially with the intention of deceiving customers. This can be done for a number of reasons, such as increasing sales, damaging the reputation of a rival, or just for one’s own benefit. Existing Machine learning models often struggle with deep contextual understanding, Scaling machine learning models while maintaining accuracy and efficiency becomes increasingly challenging as the volume of online reviews continues to grow.

Deep learning solutions have the capacity to significantly improve the accuracy and efficiency of fake review detection compared to manual techniques by automating the process. Natural language processing is one of the most widely employed methods by AI-based fake review identification systems^1^. In order to evaluate whether a review is real or fraudulent, Natural Language Processing algorithms are applied to examine the language^2^ used including the words involved, sentence structure, and sentiment. Although AI-based fake review detection systems have some promise there are still a number of issues that need to be resolved.

The necessity for a lot of high- quality training data to train the algorithms is one of the key difficulties. Systems that can deal with the dynamic nature of fraudulent reviews, which might change continuously in order to avoid detection, present another issue. In our study, we examine the effectiveness of multiple deep learning models across diverse dataset types and sizes.

The major contributions of this work are as follows:

- To address the challenges posed by existing machine learning models on deep contextual understanding, a finetuned DeBERTa is used which focus on encoding content and position of the text and it can more accurately interpret structure of the sentences.
- To enhance the detection of fake reviews ten different optimization techniques were analyzed across different datasets and the Monarch Butterfly Optimization Algorithm when combined with DeBERTa produced the superior performance by optimizing the feature selection in diverse fake review domains.
- To efficiently detect the fake reviews, a novel MBO-DeBERTa is proposed. The Monarch Butterfly Algorithm is used to optimize feature selection, while DeBERTa, a powerful language model, processes and analyses the textual data. This combination improves the accuracy and efficiency of identifying fraudulent reviews.
- To evaluate the robustness of the proposed model by detecting adversarial attacks using the Fast Gradient Sign Method (FGSM) and evaluating its resistance to such attacks and noisy data.

The manuscript is organized as follows: Section II explains the literature review in the field of fake review detection. Section III presents the methodology of the proposed model. Section IV presents the experimental results and findings of the proposed model. Section V discusses the conclusion of the work indicating future work.

## Literature survey

The detection of false reviews has emerged as a key challenge in the field of sentiment analysis^3^ as the reliance on internet reviews for product and service evaluation grows. The issue of fake review detection has been the subject of extensive research in recent years, which has used a variety of approaches, including natural language processing machine learning, and network analysis as shown in Table 1 below.

**Table 1:** Literature review.

Authors	Year	Methodology	Dataset used	Evaluation Metrics	Key Findings	Limitations
Joni Salminen, Chandrashekhar Kandpal, Ahmed Mohamed Kamel, Soon-gyo Jung, Bernard J. Jansen^4^	2022	ULMFiT (Universal Language Model Fine-tuning for Text Classification) GPT-2 (Generative Pre-trained Transformer 2)	Amazon e-commerce	Accuracy Precision Recall F1-Score Human Benchmark Comparison	The study demonstrated that machine learning models like ULMFiT and GPT-2 could generate realistic fake reviews,	The study’s limitations include a narrow dataset of Amazon reviews, limited generalization of the models to other domains, and insufficient real-world testing.
S. M. Anas S. Kumari^5^	2021	Naïve Bayes Support Vector Machines (SVM) Decision Trees	Product reviews	Accuracy Precision Recall F1-Score ROC-AUC Confusion Matrix	The potential of opinion mining and machine learning in addressing the widespread problem of fake product reviews.	These limitations suggest that while the system provides a solid foundation for fake review detection, there may be challenges in scalability, generalization, and adaptability to evolving fake reviews.
S. N. Alsubari S. N. Deshmukh A. A. Alqarni N. Alsharif T. H. Aldhyani F. W. Alsaade O. I. Khalaf^6^	2022	Support Vector Machines (SVM) Decision Trees Random Forests Neural Networks, or Logistic Regression	Reviews collected from TripAdvisor website	Accuracy Precision Recall F1-Score ROC-AUC Confusion Matrix	The supervised learning models are used in identifying fake reviews.	The study includes potential issues with dataset generalizability, these limitations can guide future research in improving the robustness, scalability, and applicability of fake review detection systems
G. S. Budhi R. Chiong Z. Wang^7^	2021	Support Vector Machines (SVM) Random Forest Naïve Bayes Logistic Regression	Yelp Dataset	Accuracy Precision Recall F1-Score Confusion Matrix	The resampling imbalanced data significantly enhances the ability of machine learning models to detect fake reviews	This study offers valuable insights into improving fake review detection through resampling techniques and machine learning, there are still challenges related to model generalization, scalability, and the evolving nature of fake review
Petr Hajek, Adela Barushka, Milan Munk^8^	2020	Deep neural, word embeddings, emotion mining	Hotel and restaurant reviews sourced from the Yelp Dataset Challenge	Accuracy Precision Recall F1-Score	This focus on integrating word embeddings and emotion mining within deep neural network frameworks	The limitations could involve incorporating a broader range of features, utilizing more diverse datasets, enhancing emotion detection techniques, improving model interpretability, and testing across various contexts to strengthen the robustness and applicability of fake review detection systems.
H. Khan M. U. Asghar M. Z. Asghar G. Srivastava P. K. R. Maddikunta T. R. Gadekallu^9^	2021	Logistic Regression Support Vector Machines (SVM) Random Forest Naive Bayes Gradient Boosting algorithms (e.g., XGBoost)	Online Textual content	Accuracy Precision Recall F1-Score	The supervised machine learning approach successfuly classifies fake reviews.	The dataset used might not fully represent the diversity of online review platforms, which can affect model generalization. Feature selection can be challenging, and poor feature choices might lead to overfitting or underfitting. Evolving deceptive tactics mean models may need frequent updates to stay effective.
L. Gutierrez-Espinoza F. Abri A. S. Namin K. S. Jones D. R. Sears^10^	2020	Bagging Boosting Stacking	Restaurant Dataset	Accuracy Precision Recall F1-Score	The study highlights the potential of ensemble learning in fake review detection, contributing to more reliable automated systems for online content verification	The limitations suggest that further research is needed to refine feature selection, enhance dataset diversity, and adapt models to evolving deceptive techniques
J. Yao Y. Zheng H. Jiang^11^	2021	Decision Tree Support Vector Machine (SVM) Logistic Regression Naive Bayes K-Nearest Neighbors (KNN)	Yelp Dataset	Accuracy Precision Recall F1-Score	Data resampling addressed class imbalance, improving the model’s ability to learn from both fake and genuine reviews.	The limitations are the use of a small dataset, which may affect the model’s generalizability, and the complexity of feature selection, which requires continuous refinement. Additionally, the model may struggle with evolving fake review tactics and can be computationally expensive due to the ensemble approach and optimization processes.
Saumya, S. Singh, J. P^12^.	2020	LSTM Autoencoder Unsupervised Learning Anomaly Detection Embedding Techniques	Amazon review dataset	Accuracy Precision Recall F1-Score REA, FPR and FNR AUC-ROC	The approach identifies spam reviews by analyzing reconstruction errors, without requiring labeled data.	The limitations of the paper include reliance on an appropriate reconstruction error threshold, which can be challenging to set. The model may struggle with detecting sophisticated spam patterns and could face scalability issues with large datasets. Additionally, the study may not have compared the approach with other unsupervised methods or tested it extensively across diverse domains.
Hassan, R. Islam, M. R^13^.	2020	Support Vector Machines (SVM) Random Forest Logistic Regression Neural Networks	Amazon dataset	Accuracy Precision Recall F1-score Confusion Matrix	The supervised machine learning is a promising approach for detecting fake online reviews, particularly when using appropriate features and models	The limitations of the paper include reliance on labeled data, which may not always be available, and challenges with class imbalance between fake and genuine reviews. Feature engineering complexity and potential overfitting may affect model performance. The approach may also struggle with detecting sophisticated fake reviews and generalizing across diverse datasets.
Wang, J. Kan, H. Meng, F. Mu, Q. Shi, G. Xiao, X^14^.	2020	SVM (Support Vector Machine) Random Forest Logistic Regression Neural Networks	Yelp Dataset	Accuracy Precision Recall F1-score	The paper demonstrates that combining multiple feature fusion with rolling collaborative training enhances fake review detection.	The paper’s limitations include reliance on feature engineering, which could affect performance with poor feature selection. Scalability issues may arise due to the computational demands of rolling collaborative training. Additionally, challenges like class imbalance, generalization to different domains, and complexity in training could limit practical application.
A. Ligthart C. Catal B. Tekinerdogan^15^	2021	Semi Supervised learning Support Vector Machines (SVM) Naive Bayes K-Nearest Neighbors (k-NN)	Yelp Dataset	Accuracy Precision Recall F1-score	These findings emphasize the potential of semi-supervised learning for improving the detection of opinion spam, particularly in situations where labeled data is scarce, making it an attractive approach for fake review detection on large platforms.	The paper’s limitations include dependence on the quality of labeled data, which can affect performance. Scalability issues arise with large datasets, and the model may struggle to generalize across domains. Additionally, class imbalance and assumptions about unlabeled data may still pose challenges.
S. Noekhah N. binti Salim N. H. Zakaria^16^	2020	The integration of graph-based methods with traditional machine learning classifiers like SVM and Naive Bayes	Synthetic crowdsourced Dataset	Accuracy Precision Recall F1-score	The multi-iterative graph-based model is a promising approach for opinion spam detection, providing more accurate results by incorporating both graph structure and traditional machine learning classifiers.	These limitations highlight challenges in terms of scalability, data quality, feature extraction, and generalization across domains, which may affect the practical application of the model in large-scale systems.
W. Liu W. Jing Y. Li^17^	2020	BiLSTM, Dense Layer	Restaurant Reviews	Accuracy Precision Recall F1-score	The BiLSTM model outperforms traditional methods like SVM and Logistic Regression in detecting deceptive reviews by capturing contextual and sequential information. Incorporating sentiment and lexical features enhances performance, improving precision, recall, and F1-score. Its bidirectional nature allows the model to detect subtle deception patterns more effectively.	The paper’s limitations include a reliance on accurately labeled data, which can affect performance if labels are inconsistent, and scalability issues due to the computational intensity of BiLSTM models. Additionally, the model may not generalize well across different domains, as deception patterns can vary.
Santhosh Vinayagamurthy^18^	2022	Transformer Models BERT	Yelp Dataset	Accuracy Precision Recall F1-score AUC-ROC MSE Confusion Matrix	These findings highlight the superior capabilities of Transformer models in handling the complexities of review deception and sentiment analysis, paving the way for more accurate and scalable detection systems in practical applications.	The major challenges regarding computational complexity, domain generalization, and data labeling quality in deceptive review detection and sentiment analysis.
Chandaka Babi et al^19^.	2023	BERT and Fine-Tuning BERT	Opinion Spam Dataset	Accuracy Precision Recall F1-score AUC-ROC Confusion Matrix	The paper demonstrates that BERT is highly effective for detecting fake online reviews due to its ability to understand the context and relationships in the text. Fine-tuning BERT improves its accuracy and generalization, making it suitable for real-world applications in fake review detection.	The limitations of the study highlight challenges related to computational cost, data requirements, interpretability and generalization when using BERT for fake review detection. These factors may restrict the model’s deployment in some real-world applications on large and diverse reviews.
Rami Mohawesh et al^20^.	2024	LSTm and RoBERTa	Yelp Review Dataset	Accuracy Precision Recall F1-score	The proposed model demonstrates improved performance in detecting fake reviews compared to traditional methods, highlighting the effectiveness of combining LSTM with RoBERTa for this task.	The model outperforms traditional methods but faces challenges such as the need for large labeled datasets, high computational complexity, and potential overfitting. Additionally, issues related to generalization across platforms, lack of interpretability, and real-time processing remain limitations of the approach.
Mian Muhammad Danyal et al^21^.	2024	BERT and XLNet	Internet Movie Database (IMDb)	Accuracy Precision Recall F1-score	The combined BERT and XLNet model outperform individual models in sentiment classification tasks, demonstrating improved accuracy and robustness in analyzing movie reviews.	The study acknowledges the need for large labeled datasets for training and the computational complexity associated with transformer-based models like BERT and XLNet.
R. A. Duma Z. Niu A. S. Nyamawe J. Tchaye-Kondi N. Jingili A. A. Yusuf A. F. Deve^22^	2024	SVM with BERT	Deceptive opinion corpus	Accuracy Precision Recall F1-score AUC-ROC Confusion Matrix	The study highlights the need for more inclusive, feature-rich, and context-aware approaches to improve fake review detection methods.	These limitations for future research to develop more robust, inclusive and context-aware fake review detection models.
Yong Pan Lijun Xu^23^	2024	K-Means DBSCAN	Face Forensics + + Dataset	Accuracy Precision Recall F1-score	These findings contribute to the field by providing an unsupervised framework for fake review detection for enhancing the reliability of online reviews in e-commerce platforms.	The method’s effectiveness is limited by challenges in feature identification, potential high false positive rates, and limited generalizability.
D. T. T. Thuy L. T. M. Thuy N. C. Bach T. T. Duc H. G. Bach D. D. Cuong^24^	2024	DenyBERT	Product Reviews	Accuracy Precision Recall F1-score	The findings suggest that DenyBERT offers an effective and reliable solution for detecting fake reviews, making it a valuable tool for maintaining the integrity of user-generated content on online platforms.	Several limitations of denybert, including its dependence on large, high-quality datasets and the need for significant computational resources. The model may also struggle with generalization across different platforms and lacks interpretability, which could hinder transparency.
E. Elakiya R. Kanagaraj T. Paturu S. D. Nivethika R. S. Kanmani^25^	2023	Random Forest, Support Vector Machine (SVM), and Naive Bayes.	Amazon Product Reviews Dataset	Accuracy Precision Recall F1-score	These findings underscore the potential of machine learning techniques, particularly the Random Forest classifier, in accurately classifying text feedback.	Several limitations, including challenges with data annotation, which can be time-consuming and prone to human errors. The risk of overfitting is another concern, as models may struggle to generalize to new data.
S. Kanmani S. Balasubramanian^26^	2023	BERT, RoBERTa, XLNet, XLM-RoBERTa	Deceptive Opinion Spam Corpus	Accuracy Precision Recall F1-score	The effectiveness of combining sentiment and readability features with advanced transformer models to enhance the detection of fraudulent reviews.	They also highlight challenges with data annotation and the potential for overfitting, which can reduce model generalization. These limitations affect the scalability and real-world applicability of their methods.
S. Khalif K. Mane^27^	2024	Machine learning and deep learning methods	Product review	Accuracy Precision Recall F1-score	They review various machine learning and deep learning techniques, emphasizing the importance of feature selection for improving detection accuracy	Limitations like the lack of sufficient behavioural features, difficulties with real-time performance, and the use of small datasets that affect model generalizability. These challenges hinder the effectiveness of fake review detection methods.

## Proposed MBO- DeBERTa model for fake review detection

**Fig. 1:**
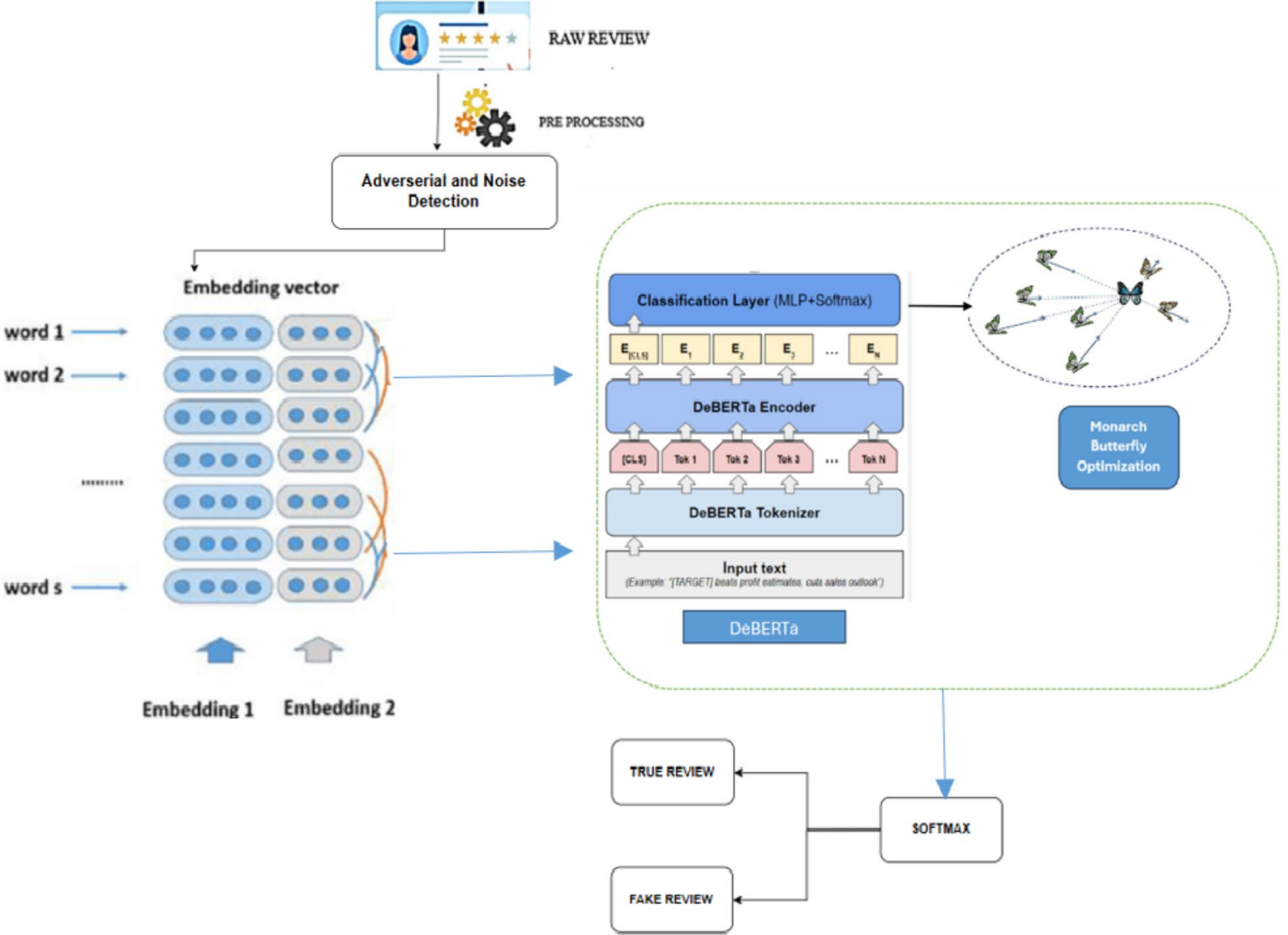
The functional diagram of the proposed MBO-DeBERTa.

DeBERTa introduces a more sophisticated version of the MLM objective, which considers both the content and position information more effectively. By separately encoding content and position, DeBERTa can more accurately interpret the structure of sentences. This helps the model to learn richer representations during pre-training and it is sent to the classification layer. Further, adoption of Monarch butterfly optimizer is used for enhancing the detection. Lastly the soft max layer is used for accurate detection of the fake review.The functional diagram of the proposed MBO-DeBERTa, model is shown in Fig. 1. The input text reviews from the dataset are preprocessed. Then they are sent to adversarial attack detection phase where it detects the noise in various levels of 10,20 and 50% thereby checking the robustness of the model. To normalize the text data, the reviews are then lemmatized, which involves reducing words to their root form. Finally, the reviews are tokenized, which means they are broken down into individual words or phrases, and embedding techniques like GloVe are used to convert the text data into numerical representations for further analysis. Then the output from the word embeddings is sent as a input to DeBERTa model. The following subsections provide the detailed description of each of these phases in the proposed model.

### Rationale for the choice of DeBERTa

DeBERTa is highly effective for fake review detection because of its advanced language modeling capabilities, which include disentangled attention mechanisms and improved positional encoding. These enhancements enable DeBERTa to generate more precise text embeddings, allowing it to detect fake linguistic patterns and anomalies thus enhances its accuracy and reliability in detecting fake reviews. DeBERTa is a Transformer-based model. DeBERTa disentangles the input into two separate vectors: one for content (token embeddings) and another for position (relative position embeddings). This permits the model to better recognize the relationship between words and their positions in a sentence, improving its contextual understanding. It uses cutting-edge features including cross-layer parameter sharing, large-scale training, and dynamic masking. This helps in distinguishing genuine reviews from fake ones, which might have unnatural phrasing or misplaced emphasis.This is given in the below Eq. (1) Whereas D is the trained DeBERTa model,

1 $$\:p\left(fake\right)\text{}=softmax\left(D\right(Tokenize\left(rnew\text{}\right)\left)\right)$$

Algorithm for the DeBERTa model is shown in Algorithm 1 below,
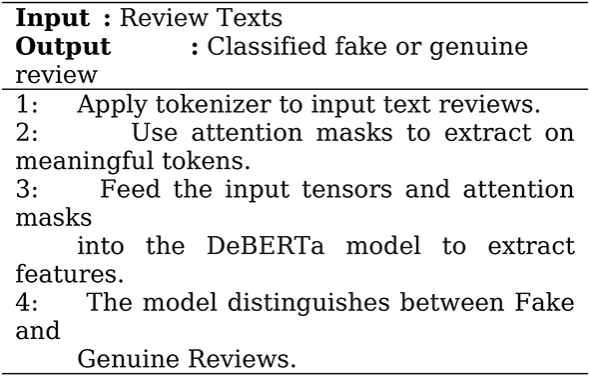


### DeBERTa word embedding mechanism

The notion of word embeddings is important for employing DeBERTa for fake review identification, however DeBERTa works differently than typical static embeddings such as Word2Vec or GloVe. DeBERTa generates contextualised embeddings, which means that the embedding for each word is determined by the complete phrase or review, rather than the word itself. These embeddings are dynamic and adapt to the surrounding context, which is critical for tasks such as fake review identification, where the tiny difference between a genuine and a false review is frequently found in how words are used in context. DeBERTa employs a disentangled attention technique to generate embeddings. This mechanism separates a word’s content from its position in the sentence, allowing the model to better capture long-range dependencies and complex contextual information, which is critical for detecting fake reviews. Unlike traditional models where word embeddings are static and represent a fixed meaning for each word, DeBERTa embeddings change based on how the word interacts with other words in the review.

DeBERTa uses both the content embedding $\:{V}_{{W}_{C}}$and$\:\:{V}_{{W}_{p}}$ for each word. The final embedding for a word w is a combination of these two as shown in Eq. (2) below,

2 $$\:{V}_{w}\:=Attention({V}_{{W}_{C}}\:,{V}_{{W}_{p}},Context\:)$$

This disentangled attention mechanism allows the model to focus not only on the word’s meaning but also on how it is used in the sentence, which is crucial for detecting nuanced patterns in fake reviews. The review is tokenized and passed through multiple attention layers, where each word is assigned a dynamic embedding based on its relation to other words.

The core of DeBERTa’s embedding relies on the multi-head self-attention mechanism. The attention score for a word pair ($\:{W}_{i},{W}_{j}$)is computed as shown in the below Eq. (3) below,

3 $$Attention\,(\:{W}_{i},{W}_{j})=Softmax\,\:(\:\:\frac{{(V}_{{Q}_{wi}}){{(V}_{{K}_{wj})}}^{T}}{\sqrt{{d}_{k}}})\cdot\:{V}_{{v}_{wj}}$$

In DeBERTa, this disentangled attention refines the embeddings of words based on both their content and positional information, allowing the model to better understand complex dependencies that might indicate deceptive language in fake reviews.

### Rationale for the choice of MBO

Monarch Butterfly Optimization (MBO) is an exact choice for fake review detection due to its efficient and adaptive search capabilities, which are crucial for optimizing hyperparameters and feature selection in complex datasets. MBO’s ability to balance exploration and exploitation ensures robust performance even with noisy and variable review data, while its global optimization process helps to avoid local optima and achieve superior model accuracy. These characteristics make MBO a powerful algorithm for enhancing the effectiveness of fake review detection. The migratory patterns of monarch butterflies serve as the inspiration for the Monarch Butterfly Optimization (MBO) algorithm, which is used to detect fake reviews. MBO enhances this process by optimizing feature selection and tuning model parameters^28^. It uses a combination of global search (migration operator) and local search (butterfly adjustment operator) to identify relevant features and fine-tune transfer learning models, improving their accuracy and reliability. This is expressed in Eq. (4) to(7).

4 $$X=\left\{X1, X2,....,XN\right\}$$

Where N is the number of butterflies

5 $$\:{x}_{i}^{new}=\:{x}_{j}\:for\:i\in\:X1$$

6 $$\:{x}_{i}^{new}=\:{x}_{i}\:+{r}_{i}\:.\:({x}_{best}-{x}_{i})\:$$

7 $$\:f\left({x}_{i}^{new}\right)\:\:for\:each\:\:{x}_{i}^{new}$$

This algorithm starts by initializing a population of candidate solutions, each denoted as a vector in a D-dimensional space. The search space is divided into two sub-populations, L1 and L2, with their sizes determined by a partition ratio. Butterflies migrate between these sub-populations based on their fitness, where the new position of a butterfly xi in sub-population X1 is set to xj from another butterfly. Each butterfly’s position is then adjusted towards the best solution found so far using a formula that incorporates a random factor ri. This process of migration and adjustment continues iteratively until the termination criteria are met, with fitness values evaluated for each updated solution. Algorithm 2 below illustrates the Monarch butterfly optimization algorithm.
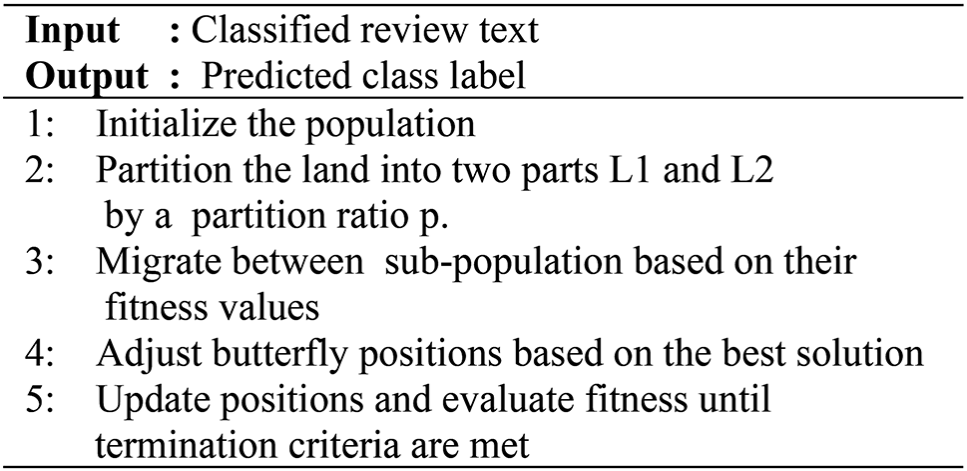


### BERT + EMBEDDING

For fake review detection, large sentence embeddings are generated using the BERT pre-trained model. The process begins by tokenizing a sentence Si into words W_o_={w_o_1, w_o_2, w_o_3…, w_o_n} with each word w_o_∈W_o_ fed into BERT to attain its corresponding embedding, such as E1 for w_o_1. This is repeated for all n words in Si, resulting in Vn embeddings. These embeddings are then united to form a large embedding for the sentence Si, represented as BE = {E1 ∪E2 ∪⋯∪En}. Once all large embeddings and their associated labels are generated for the dataset, they are loaded into classification models to detect fake reviews.

### MBO-DeBERTa algorithm

The MBO-DeBERTa is the novel method developed to solve the problems associated with precisely identifying and differentiating between genuine and fake reviews. This algorithm process starts by splitting the dataset into training, validation, and test sets. The pre-trained DeBERTa model is initialized, and its output features are used as the basis for optimization. A population of candidate solutions (monarch butterflies) is randomly generated, with parameters such as migration ratio, period, adjusting rate, and maximum step size set. The algorithm then iterates by sorting solutions based on their fitness in fake review detection, dividing them into two sub-populations. Positions of butterflies are updated based on fitness values, with migration and adjustment rules guiding their movement.

Butterflies are adjusted using local and global solutions from DeBERTa’s feature outputs, and their effectiveness is continually evaluated. This iterative process continues till the maximum number of iterations is reached, with the best solution for detecting fake reviews being returned. MBO optimize DeBERTa’s performance by efficiently searching the hyperparameter space. Instead of relying on exhaustive methods like grid or random search, MBO uses a surrogate model to predict the performance of different configurations based on prior trials. It prioritizes testing promising hyperparameter combinations, saving time and resources. For DeBERTa, MBO focuses on optimizing parameters critical to its advanced architecture, such as disentangled attention mechanisms, improving accuracy and computational efficiency. The proposed algorithm MBO-DeBERTa is given in Algorithm 3 below,
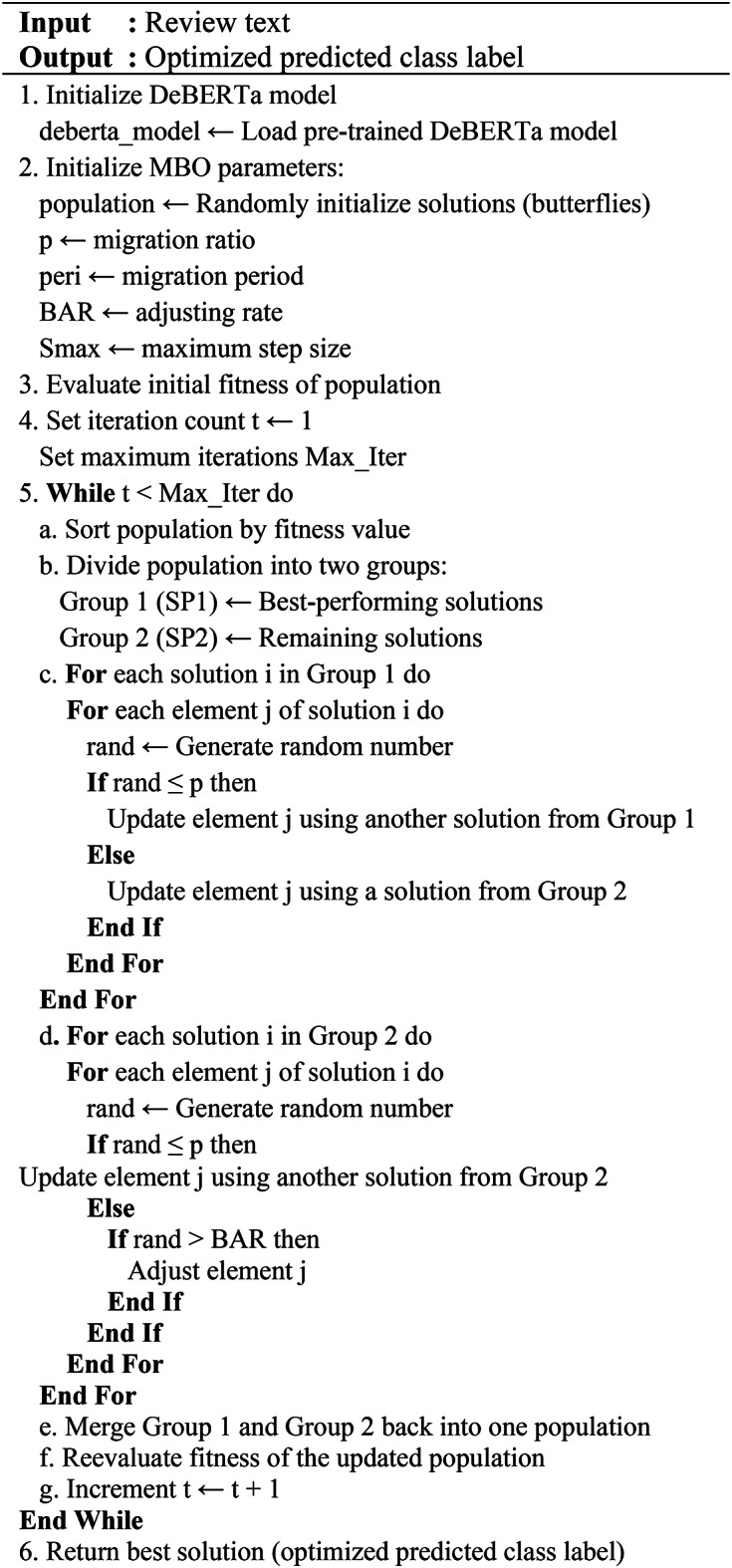


## Results and discussion

These findings include the performance of the suggested MBO-DeBERTa model, as well as a comparison section to verify the suggested system’s suitability for classifying Fake reviews.

### System configuration

This section offers a thorough examination of the performance of the suggested system, which was simulated using Python. The system was equipped with 1 TB of memory for handling large-scale computations. For AI and deep learning tasks the system features two NVIDIA H100 GPUs, each with 80GB of memory, connected via an NVIDIA NV Link Bridge to enhance GPU-to-GPU communication. The system runs on Ubuntu 24.04 LTS, ensuring stability and compatibility for high-performance computing applications.

**Table 2:** Dataset description.

Datasets	Size	No of Fake Reviews	No. of Real Reviews	Polarity	Avg Review length (words)
Amazon fake review^29^	21,000	10,500	10,500	Positive, negative and neutral	67.465
Fake review^30^	40,000	20,000	20,000	Positive, negative and neutral	69.192
Deceptive Opinion Spam Corpus^31^	1,600	800	800	Positive and negative	148.775

**Table 3:** Training and testing Split up.

Datasets	Augmented Size	Size of Training Data after 80% − 20% train – test split	Size of Test Data after 80% − 20% train – test split
Amazon fake review^29^	21,000	16,800	4,200
Fake review^30^	40,432	32,346	8,086
Deceptive Opinion Spam Corpus^31^	16,000	12,800	3,200

### Dataset description

This study includes three distinct datasets as given in Table 2. The fake review dataset by Joni Salminen et al. includes 40,000 reviews split between real Amazon reviews and AI-generated fake reviews, useful for assessing fake review detection models. The Deceptive Opinion Spam corpus dataset contains hotel reviews of 1600 collected from various sources, including genuinely and falsely positive and negative reviews, adding realism through Mechanical Turk-generated fakes. To address the small size of the deceptive spam corpus dataset (1600 reviews) and enhance model robustness, data augmentation techniques such as GPT-Based Augmentation^32^ was applied involving GPT-4 to paraphrase and modify reviews while preserving their meaning, creating diverse variations that mimic real-world text. This helped the model recognize different syntactic structures and strengthened its resistance to AI-generated fake reviews. In addition it also utilized Word Embedding Perturbation^33^ technique pretrained with Word2Vec embeddings to replace words with semantically similar counterparts, modifying nouns, adjectives, and verbs while preserving meaning. This technique improved the model’s ability to detect deceptive reviews with slight modifications, making it more resilient to word-level adversarial attacks. These data augmentation techniques not only helped to expand our dataset from 1600 to 16000 while maintaining semantic consistency and diversity making it more effective in real-world fake review detection scenarios.The Amazon fake review dataset on Kaggle, with 21,000 reviews equally divided between real and fake, is relevant for evaluating detection programs in e-commerce, influencing consumer decisions and allowing for comprehensive model assessments. The fake review and Deceptive opinion spam corpus has been augmented and upscaled as it had lower reviews for achieving better results. The 80 − 20 split-up ratio is considered as allocating 80% of the dataset to training provides sufficient examples for the model to capture patterns, relationships, and features. It minimizes the risk of underfitting, which can happen if the model doesn’t see enough data. From 80% training itself 10% has been taken for validation. The remaining 20% is used for testing, offering a reasonable sample size to evaluate the model’s performance. A smaller testing set, such as in a 90 − 10 split, might lack sufficient diversity for robust evaluation, leading to unreliable performance metrics, while a larger testing set, like in a 70 − 30 split, reduces the data available for training, potentially weakening the model’s learning capacity^34^. The dataset descriptions are given in Tables 2 and 3. The Sample Dataset is given in Table 4 below.

**Table 4:** Sample reviews.

Labels	Review Text	Important Sentence(s)	Dataset
0	This bracelet is gorgeous, but I found it so uncomfortable. I don’t have large wrists, but my wrist bones do kind of stick out. No matter were I placed the cuff, it was hitting my wrist bones. I had to return it.	This bracelet is gorgeous, but I found it so uncomfortable.	Amazon^29^
1	My poster was a little wrinkled, but ill live with it. Not that big of a deal. But wish they packaged it better.	My poster was a little wrinkled, but ill live with it.But wish they packaged it better.	Amazon^29^
1	While I am not a super technical battery expert, I did want a charger that showed me some statistics on my batteries such as voltage and capacity, as well as being easy to use, and also able to handle both 18,650 and NiMH.	Everyone seems to favor the Opus products, and while I am sure they are very good chargers, the AccuPower seemed easier to use, and I have not been disappointed thus far.	Fake Review^30^
0	Two months of use and it has kept the light on for about a week. I have one other light that I had to replace, but it is still working fine. The light is nice and bright. It is not waterproof so it will need some protection.	The light is nice and bright. It is not waterproof so it will need some protection.	Fake Review^30^
1	First room’s heating system sounded like we had a generator in our room. Second room had stool in the toilet and a smear on the linens (which we didn’t notice until morning-eeewwhh! ).	First room’s heating system sounded like we had a generator in our room. Second room had stool in the toilet and a smear on the linens (which we didn’t notice until morning-eeewwhh! ).	Deceptive Opinion Spam Corpus^31^
0	My husband and I stayed at the James Chicago Hotel for our anniversary. This place is fantastic! We knew as soon as we arrived we made the right choice! The rooms are BEAUTIFUL and the staff very attentive and wonderful!!	This place is fantastic! We knew as soon as we arrived we made the right choice! The rooms are BEAUTIFUL and the staff very attentive and wonderful!!	Deceptive Opinion Spam Corpus^31^

### Performance metrics of the proposed system

The experimental evaluation of the proposed method is conducted using various metrics such as accuracy, precision, recall, F1 score. Performance measures are determined using the formulas in Eqs. (8–11).

$$\:Accuracy\:=\:\:\:(TP+TN)/(TP+TN+FP+FN)\:\:\:\:\:\:\left(8\right)$$

$$\:Precision\:=\:\:TP/(TP+FP)\:\:\:\:\:\:\:\:\:\:\:\:\:\:\:\:\:\:\:\:\:\:\:\:\:\:\:\left(9\right)$$

$$\:Recall\:=\:\:TP/(TP+FN)\:\:\:\:\:\:\:\:\:\:\:\:\:\:\:\:\:\:\:\:\:\:\:\left(10\right)$$

$$\:F1-Score\:=\:\:2.\:\:(P.R)/(P+R)\:\:\:\:\:\:\:\:\:\:\:\:\:\:\:\:\:\left(11\right)$$

Where P represents Precision and R represents Recall measure. The true positives, true negatives, false positives, and false negatives are allocated to the TP, TN, FP, and FN parameters respectively.

### Experimental results of the proposed system

**Fig. 2:**
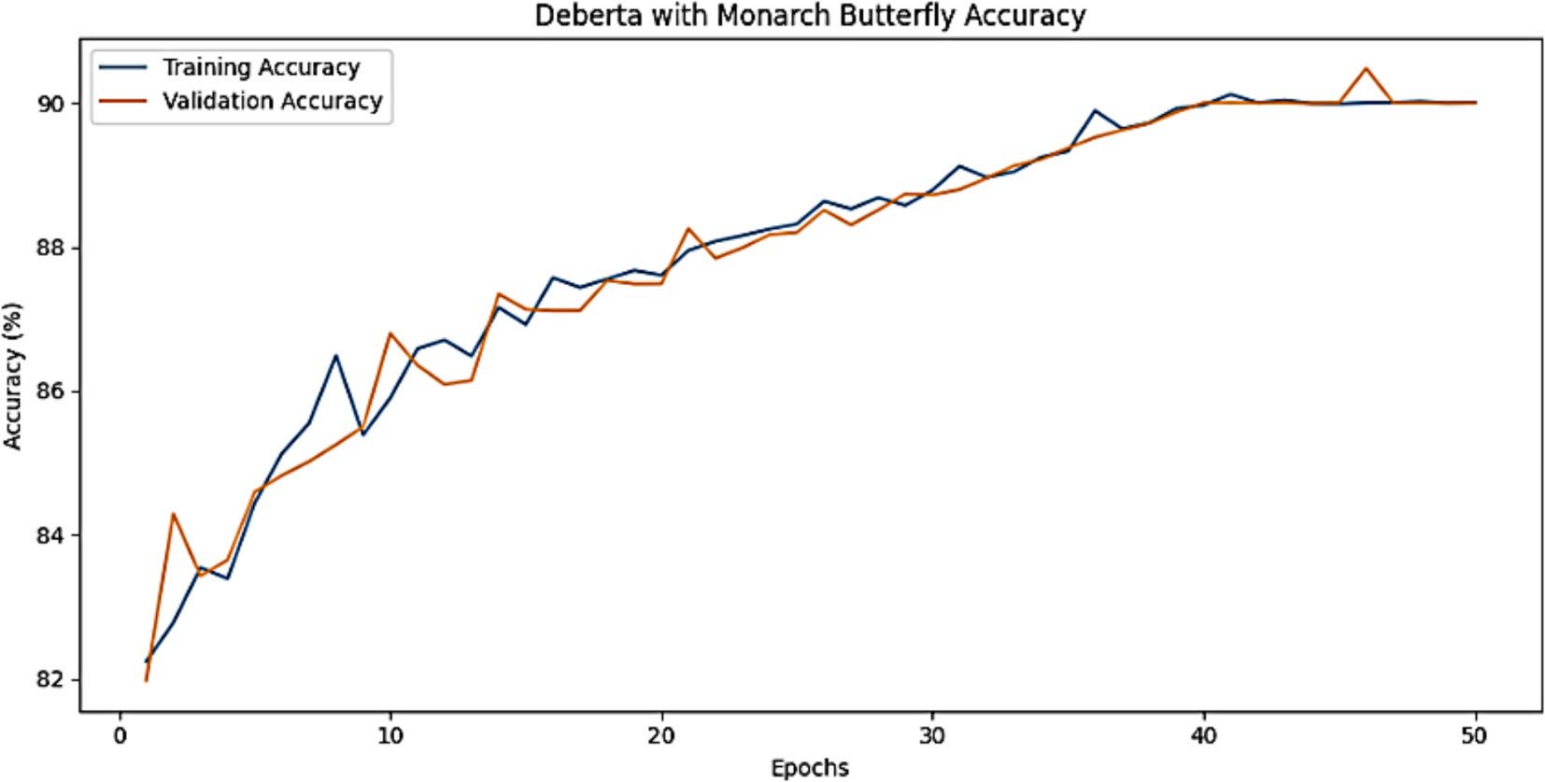
Accuracy of the proposed MBO-DeBERTa model for fake review dataset.

**Fig. 3:**
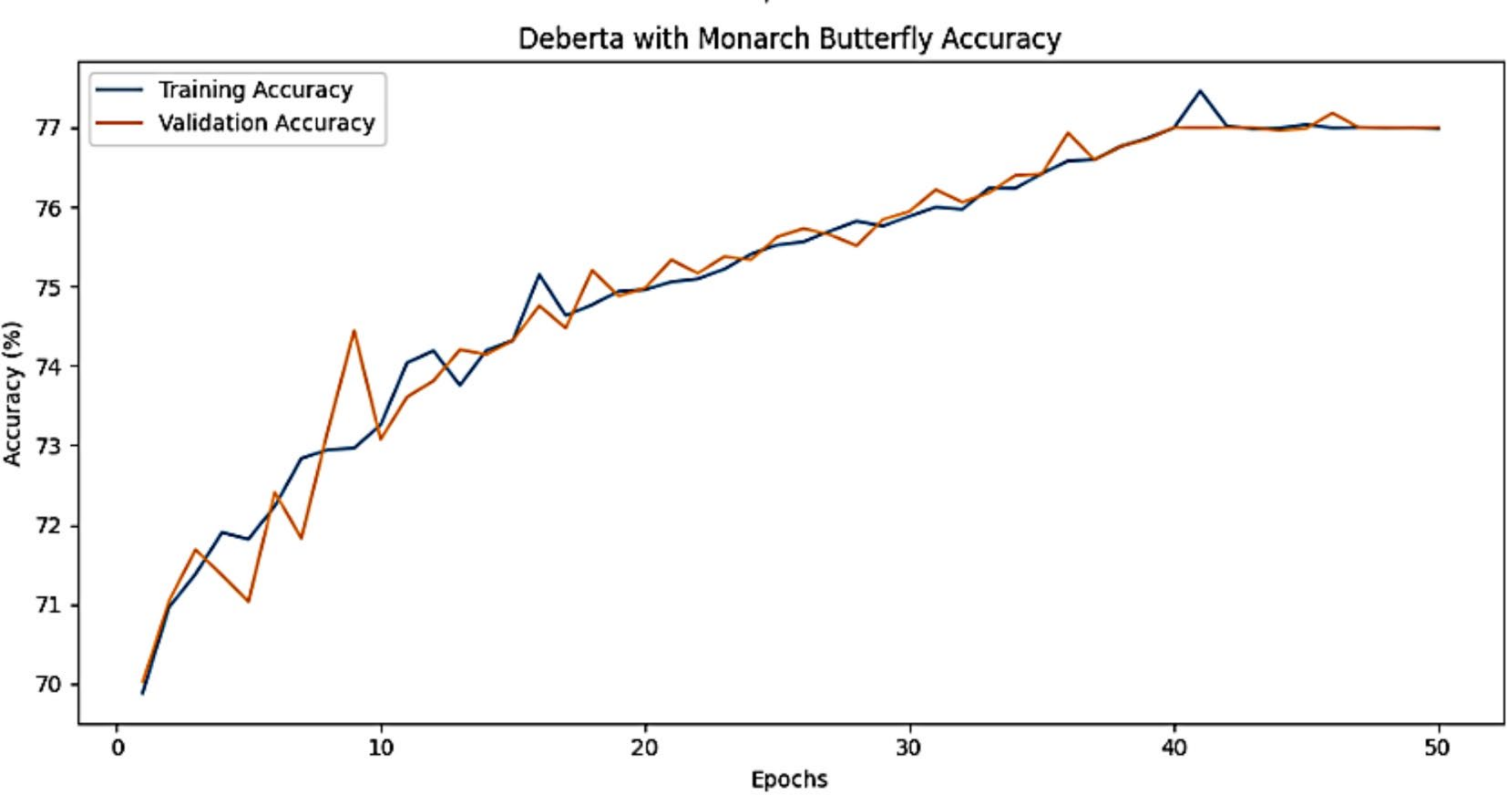
Accuracy of the proposed MBO-DeBERTa model for Amazon review dataset.

**Fig. 4:**
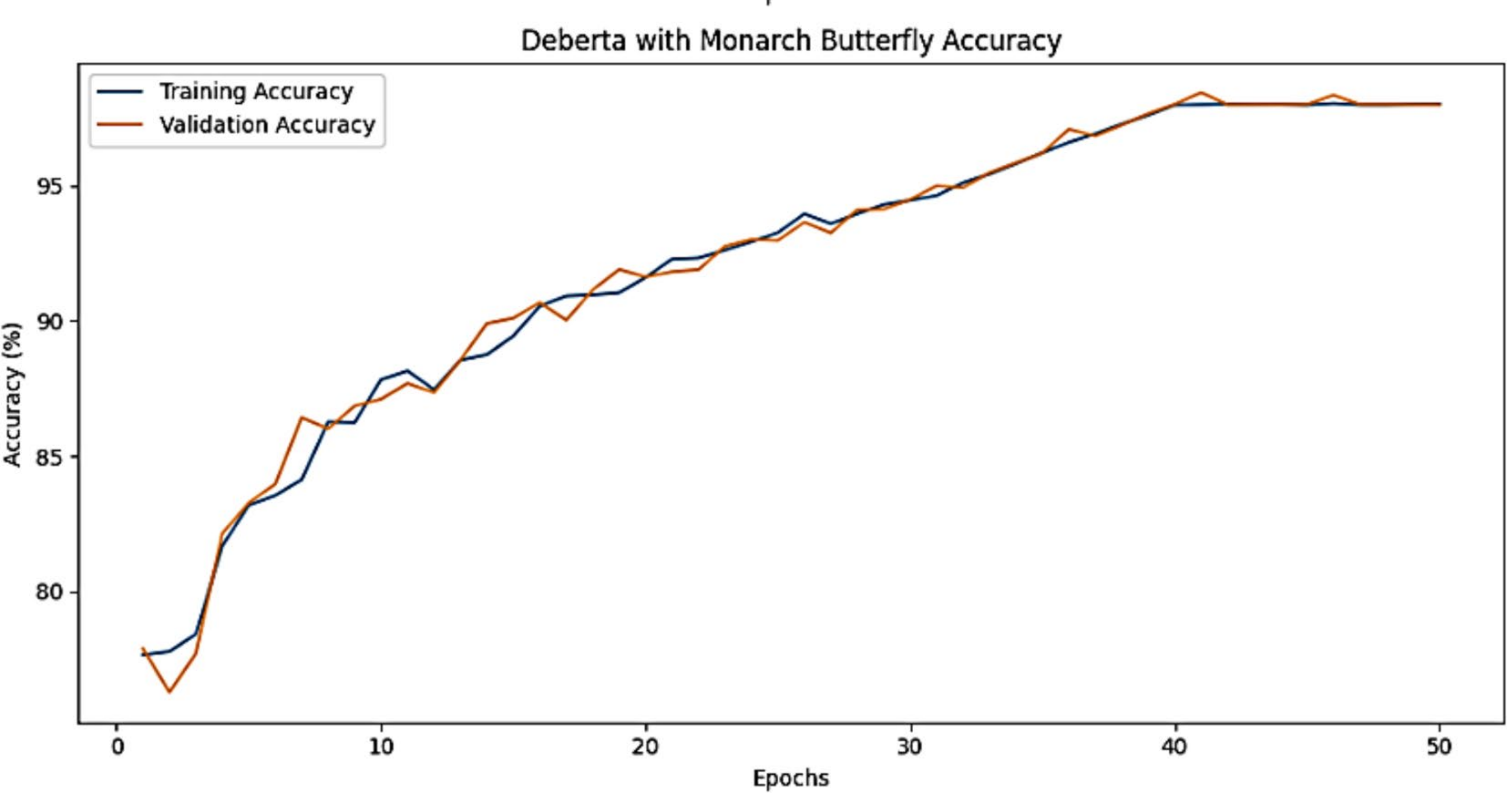
Accuracy of the proposed MBO-DeBERTa model for Deceptive Opinion review dataset.

**Fig. 5:**
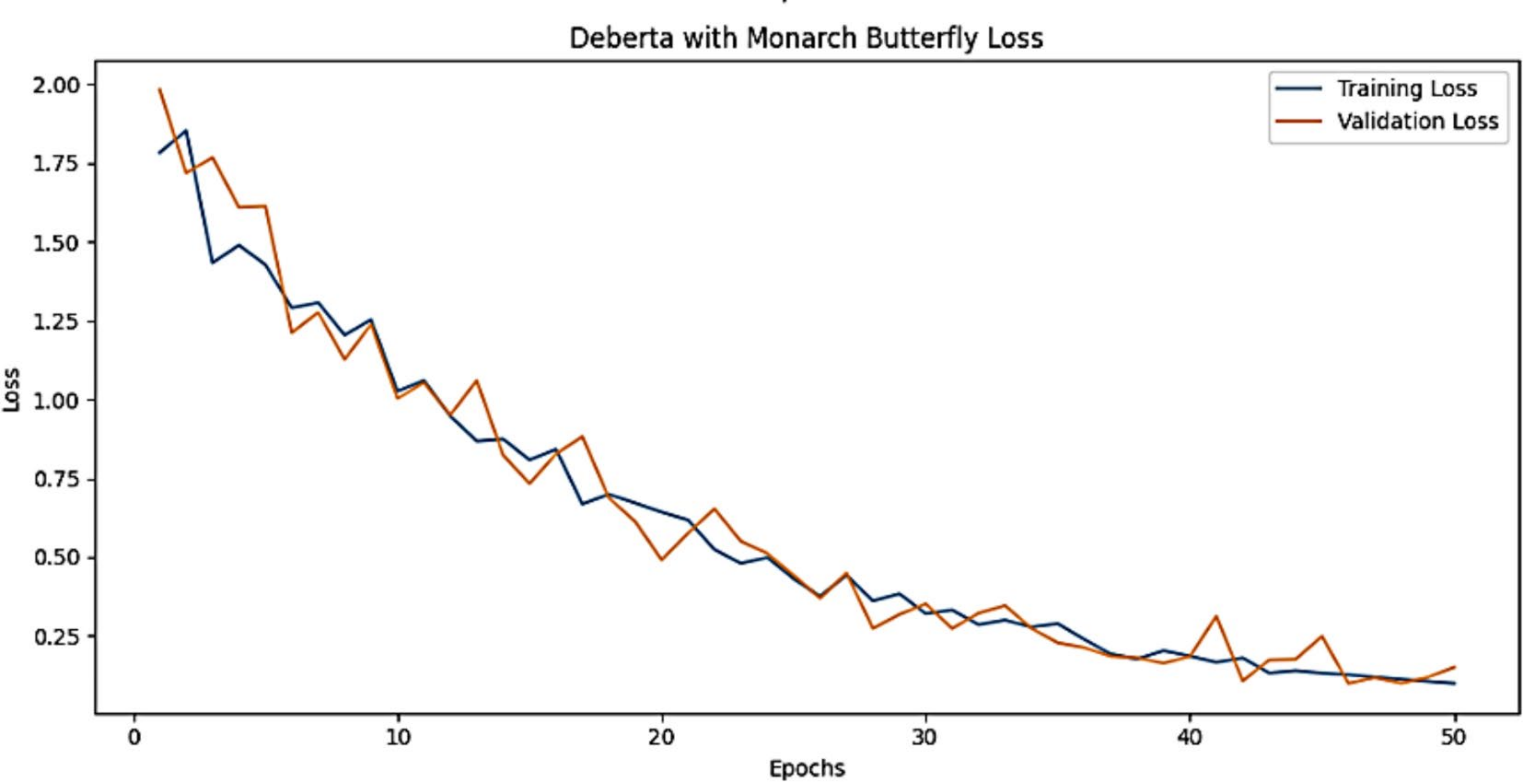
Loss curve of the proposed MBO-DeBERTa model for fake review dataset.

**Fig. 6:**
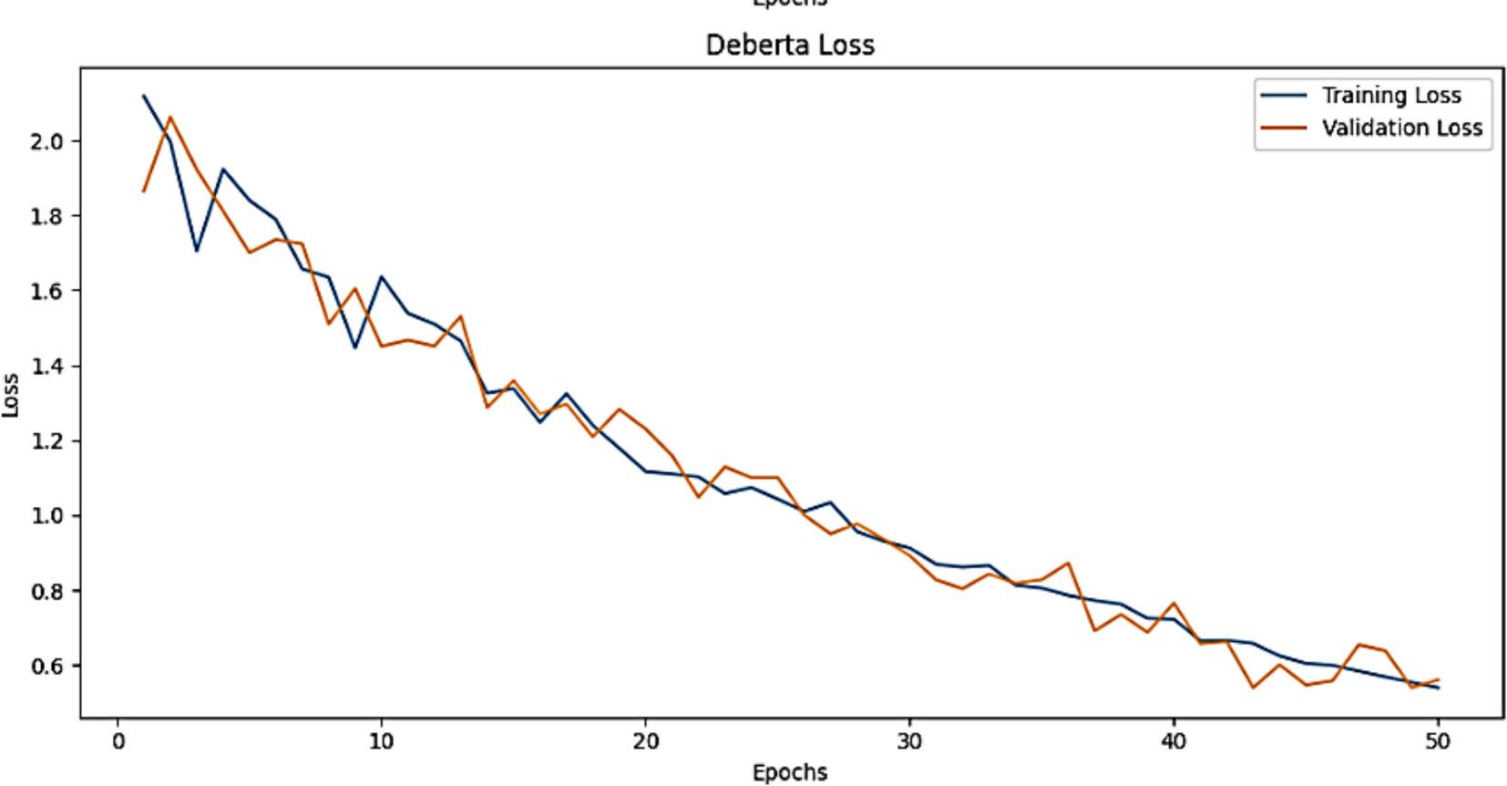
Loss curve of the proposed MBO-DeBERTa model for Amazon review dataset.

**Fig. 7:**
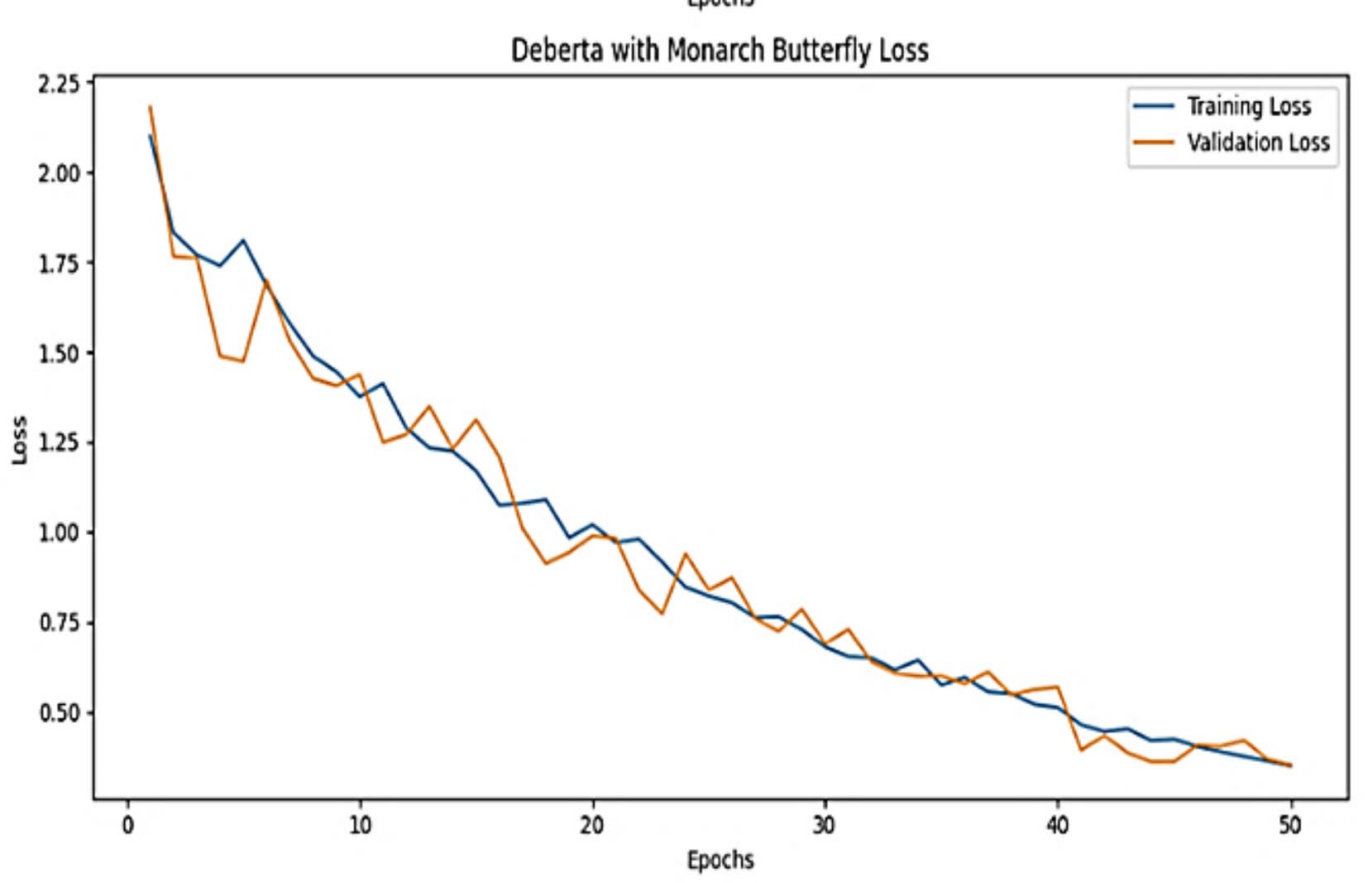
Loss curve of the proposed MBO-DeBERTa model For Deceptive Opinion Spam review dataset.

The training accuracy and testing accuracy of the suggested model with different epochs is shown in Figs. 2, 3 and 4. The analysis shows that the accuracy increases as the epoch increases. The training set obtains a maximum accuracy of 83%, 94% and 99% and the testing set achieves a maximum accuracy of 78%, 90% and 99% for the Amazon, Deceptive opinion SPam and Fake review dataset respectively. By combining positional information about review text structure from DeBERTa and improving fitness of new solution by MBO, the proposed algorithm effectively differentiates among genuine and fake reviews, thereby improving the accuracy of Fake review detection system. The training loss and testing loss rate of the suggested model with different epochs is shown in Figs. 5, 6 and 7. The analysis shows that the loss decreases as the epoch increases. When the epoch value is 50, the training set achieves a minimum loss of 0.17%, 0.6% and 0.01% and the testing set achieves a minimum loss of 0.22%,0.10 and 0.01 for the Amazon, Deceptive Opinion Spam and Fake review dataset respectively.

**Fig. 8:**
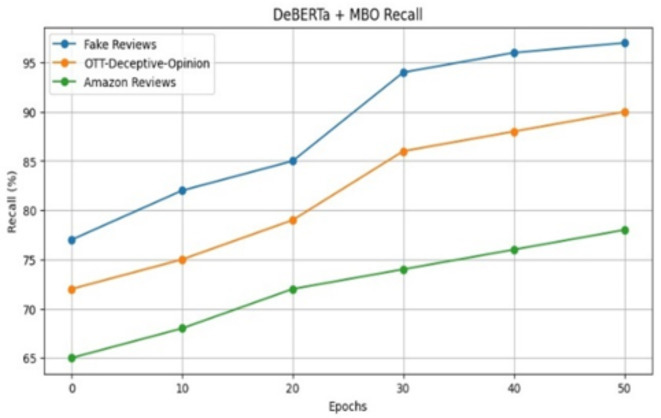
Recall of the proposed model for review datasets.

The suggested model’s recall performances are shown in Fig. 8 with varied epoch and dataset. It obtains a minimum recall value of 64% and a maximum recall value of 99%.By effectively finding the best fitness value which is crucial for review classification, MBO model reduces false negatives thus improving recall performance.

**Fig. 9:**
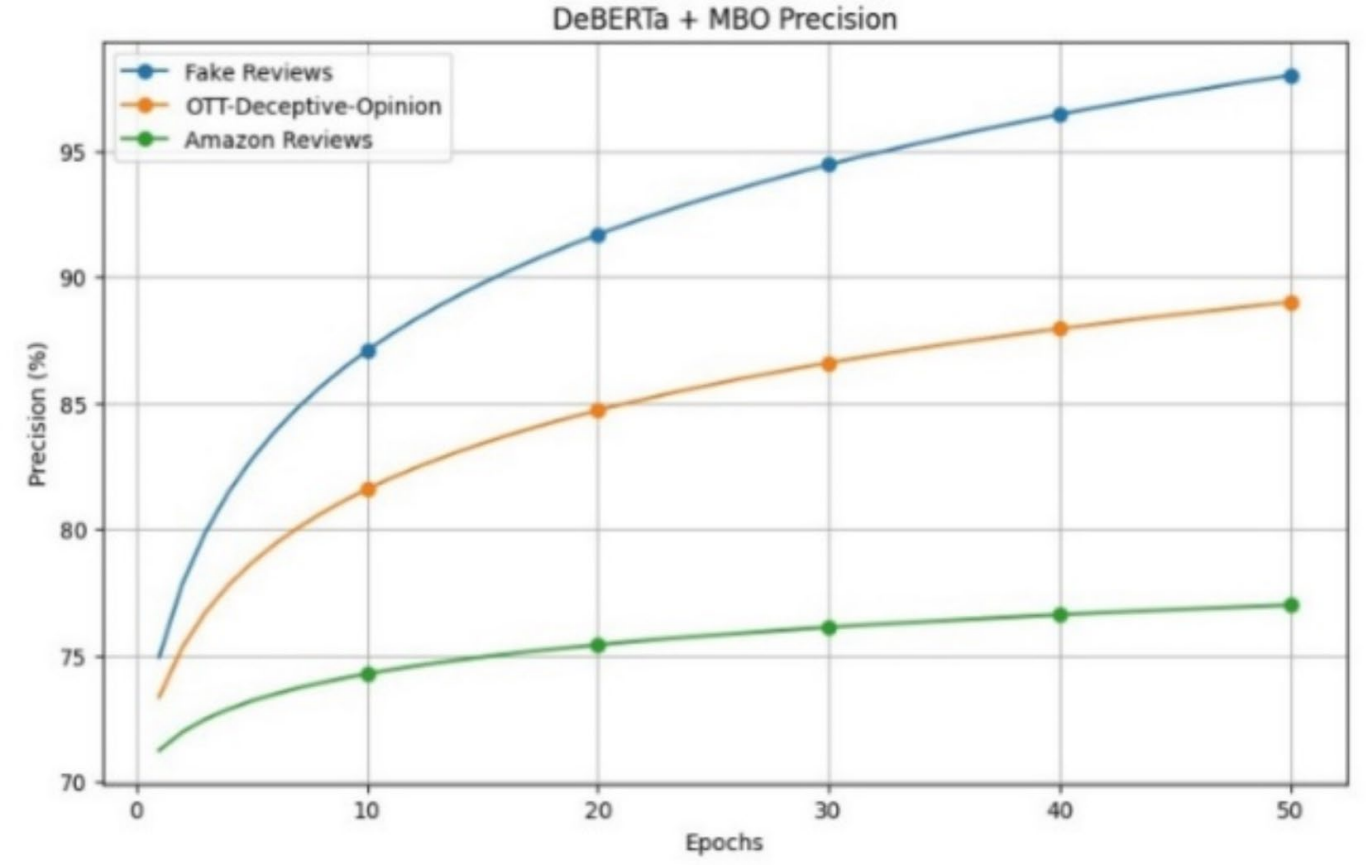
Precision of the proposed model for review datasets.

The precision performances with different epochs for the proposed model are shown in Fig. 9. The minimum precision value is 63% and the maximum precision value is 98%. DeBERTa’s precise identification of the positional encoding representation across the review context enables a more comprehensive comprehension of the features found in the review text, which improves precision.

**Fig. 10:**
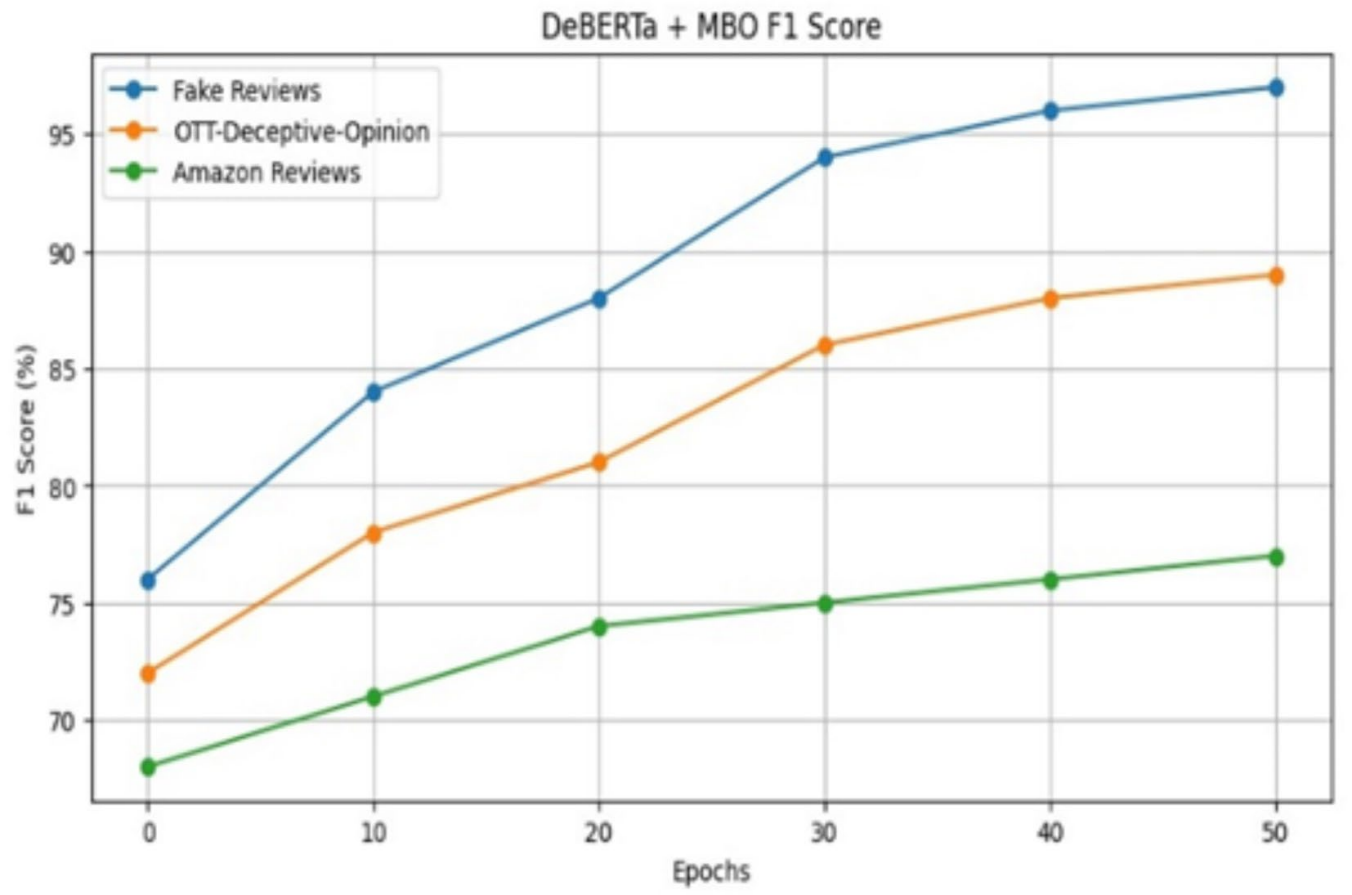
F1 Score of the proposed model for review datasets.

**Fig. 11:**
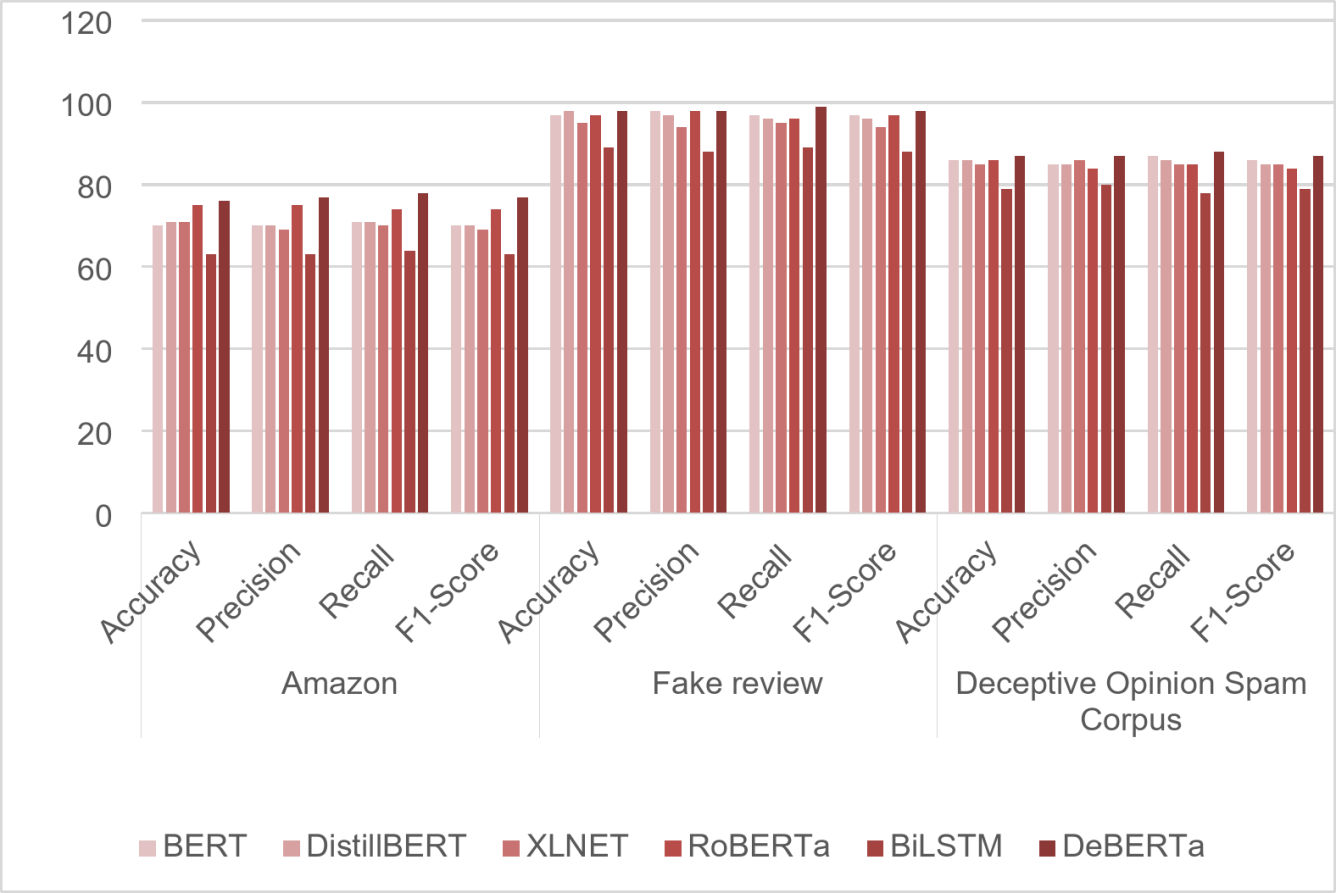
Performance Analysis of the Transformer models for three review datasets.

Figure 10 displays F1 score performances of the proposed model throughout a range of epochs. It achieves the maximum F1 score of 98% and the minimum F1 score of 63% .By merging positional data describing the review text structure from DeBERTa and enhancing the new solution’s fitness using MBO, the proposed model MBO-DeBERTa improves its ability to accurately classify fake reviews leading to an increase in F1 score. A metric-based comparison of the effectiveness of the various transformer models is shown in Table 5. Accuracy, precision, recall and F1 score are all compared and DeBERTa excels in all metrics over three distinct different datasets as shown in Fig. 11.

**Table 5:** Performance analysis of transformer models.

	Amazon				Fake review				Deceptive Opinion Spam Corpus			
	Accuracy	Precision	Recall	F1-Score	Accuracy	Precision	Recall	F1-Score	Accuracy	Precision	Recall	F1-Score
BERT	70	70	71	70	97	98	97	97	86	85	87	86
DeBERTa	76	77	78	77	98	98	99	98	87	87	88	87
DistillBERT	71	70	71	70	98	97	96	96	86	85	86	85
XLNET	71	69	70	69	95	94	95	94	85	86	85	85
RoBERTa	75	75	74	74	97	98	96	97	86	84	85	84
BiLSTM	63	63	64	63	89	88	89	88	79	80	78	79

**Fig. 12:**
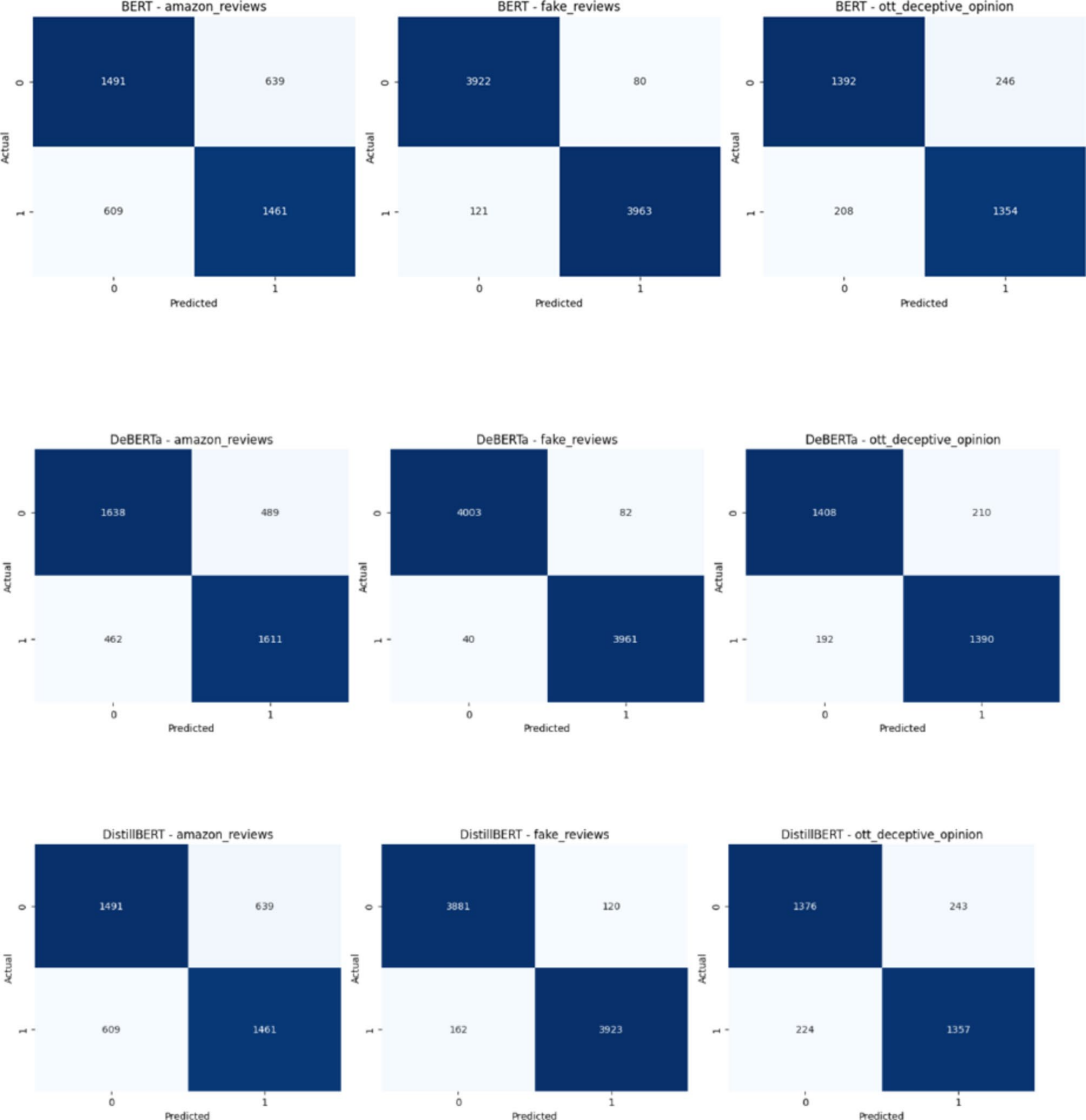

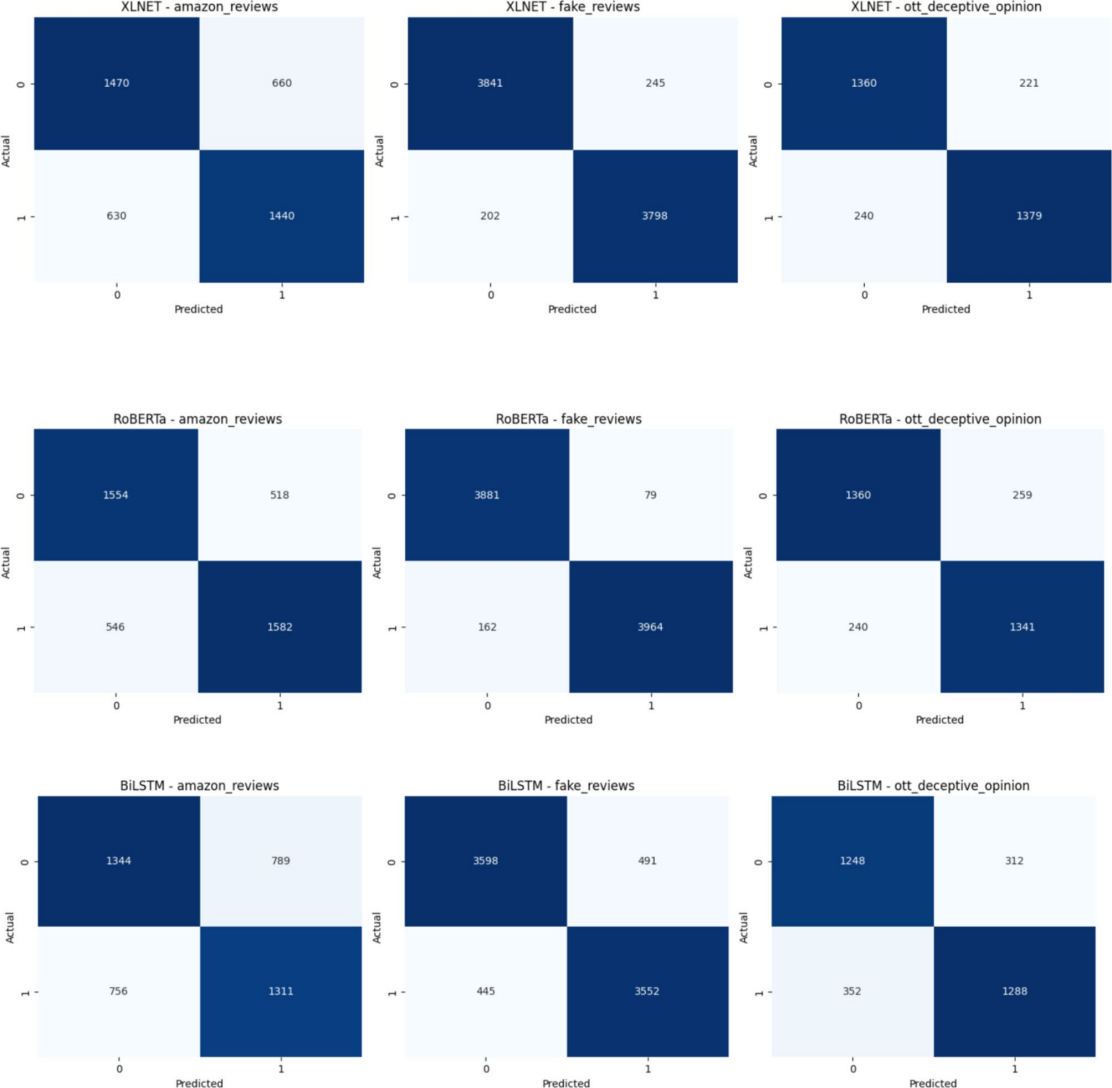
Confusion Matrix of the Transformer models for three review datasets over Augmented Review Data with 80% training and 20% testing.

**Fig. 13:**
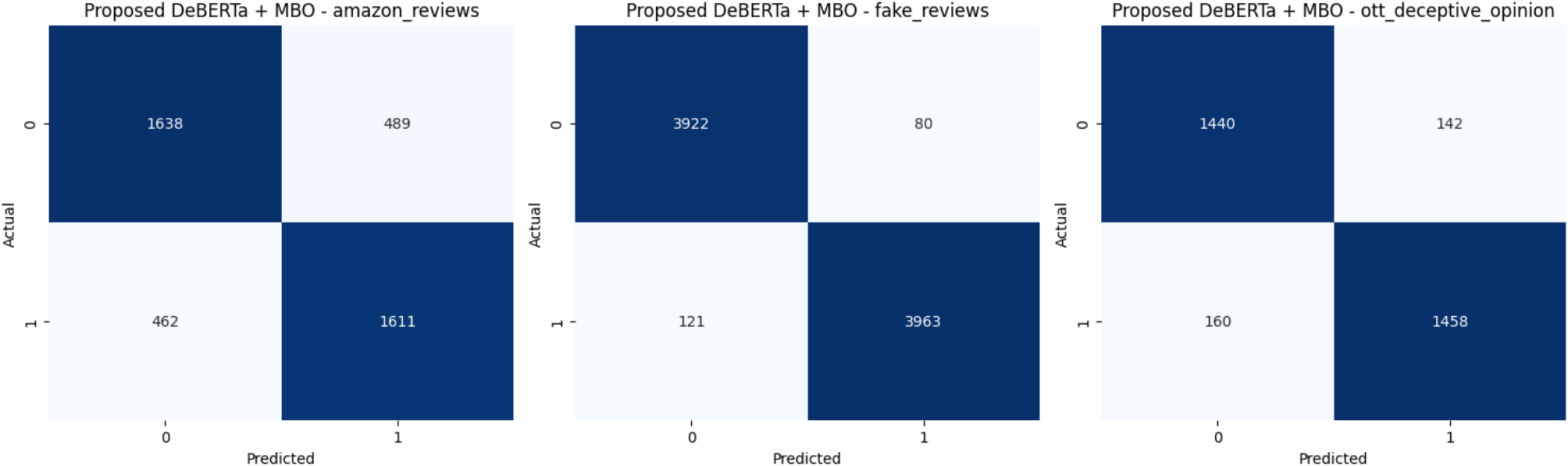
Confusion Matrix of the Proposed model for three review datasets over Augmented Review Data with 80% training and 20% testing.

**Table 6:** Comparison analysis of the proposed model.

Models	Amazon				Fake review				Deceptive Opinion Spam			
	Accuracy	Precision	Recall	F1-Score	Accuracy	Precision	Recall	F1-Score	Accuracy	Precision	Recall	F1-Score
Harris Hawks + DeBERTa	75	75	73	74	96	96	93	96	87	89	88	88
Grey Wolf + DeBERTa	73	74	72	75	96	95	93	95	89	87	86	88
Moth Flame + DeBERTa	68	68	66	67	83	81	84	82	83	81	84	82
FireFly + DeBERTa	68	65	67	66	86	86	85	85	90	88	87	87
Particle Swarm + DeBERTa	75	74	73	74	91	90	91	90	87	86	85	85
Lion + DeBERTa	68	63	67	65	82	80	82	81	82	80	82	81
Cuckoo Search + DeBERTa	68	67	69	68	85	84	83	83	78	78	79	78
Bat + DeBERTa	63	62	60	61	65	66	67	66	70	69	68	68
Bees + DeBERTa	61	61	60	60	63	64	64	64	69	67	68	67
**MBO** **+DeBERTa**	**78**	**77**	**78**	**77**	**98**	**98**	**97**	**97**	**91**	**91**	**90**	**90**

**Table 7:** Ablation study.

Dataset Used	Model/Configuration	Optimizer Used	Metrics				Training Time for 50 epochs	Avg Inference Time (per review)
			Accuracy	Precision	Recall	F1		
Amazon Reviews	DeBERTa (Baseline)	No Optimizer	76	77	78	77	36,018.10 s	0.0422 s
Amazon Reviews	DeBERTa + MBO (Proposed)	Monarch Butterfly Optimization	78	77	78	77	25,200.12 s	0.0289 s
Fake Review	DeBERTa (Baseline)	No Optimizer	98	98	97	97	60,000.25 s	0.0589 s
Fake Review	DeBERTa + MBO (Proposed)	Monarch Butterfly Optimization	98	98	99	98	50,400.32 s	0.0304 s
Deceptive Opinion Spam corpus	DeBERTa (Baseline)	No Optimizer	87	87	88	87	4132.30 s	0.0378 s
Deceptive Opinion Spam corpus	DeBERTa + MBO (Proposed)	Monarch Butterfly Optimization	91	91	90	90	2931.15 s	0.0215 s

**Fig. 14:**
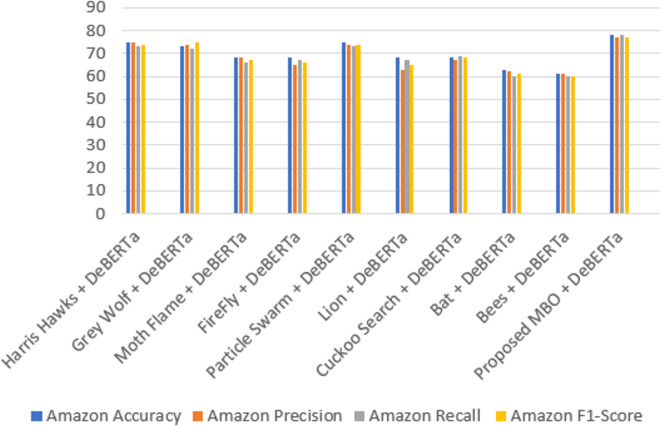
Overall comparison of the proposed model with the existing methods for Amazon review dataset.

**Fig. 15:**
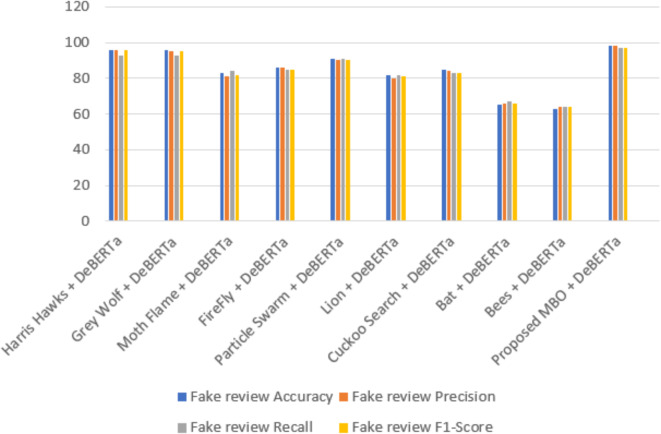
Overall comparison of the proposed model with the existing methods for Fake review datase.

**Fig. 16:**
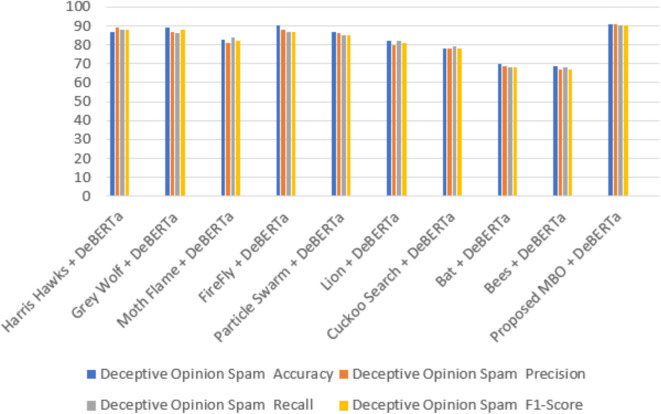
Overall comparison of the proposed model with the existing methods for Deceptive opinion review dataset.

The Figs. 12 and 13 depicts the confusion matrix for the classification results. It shows the True and Predicted labels for all three review datasets. Table 6shows a metric based comparisons with combination of DeBERTa since DeBERTa model exhibits superior performance than other transformer models and the current optimization models including Harris Hawks^35^, Grey Wolf^36^, Moth Flame^37^, FireFly^38^, Particle swarm^39^, Lion^40^, Cuckoo search^41^, Bat^42^, Bees^43^ and proposed MBO. Compared with existing models the proposed model achieves a high accuracy of 78%, 98% and 91% for Amazon, Fake review and Deceptive Opinion Spam dataset. In comparison to current models, the proposed model attains a maximum precision rate of 77%, 98% and 91% for Amazon, Fake review and Deceptive Opinion Spam dataset. The recall of the suggested model is compared to that of the current models, high recall values of the proposed model are 78%,97%and 90% for Amazon, Fake review and Deceptive Opinion Spam dataset. The suggested model receives a maximum F1 score of 77%,97% and 90% when compared to the current models. The proposed model achieves 0.22% loss rate compared with the existing models. The overall contrast between the suggested model and the current models is shown in Fig. 14 for Amazon review dataset, Fig. 15 for Fake review dataset and Fig. 16 for Deceptive Opinion Spam Review dataset. The suggested model outperforms the current model including combination of DeBERTa and the various optimization models such as Harris Hawks, Grey Wolf, Moth Flame, FireFly, Particle swarm, Lion, Cuckoo search, Bat and Bees in terms of several metrics. In Table 6 we attempted to explore if its performance can further be boosted by ensemble with optimization algorithms. In Table 7 we attempted to explore the ablation study over the usage of our proposed model. The proposed MBO + DeBERTa model produces the best performance than all other ensemble models. The proposed model was also tested on the unseen data of Indian Online Fashion store verified customer reviews which is given in the second page of the website link provided and our model works efficiently for real world data as shown below in Tables 8 and 9. As per the links provided below the reviews are classified as Verified and Non-Verified purchase. A “verified purchase” signifies that a customer bought a product from a particular platform and their review is authenticated as coming from an actual buyer. In contrast, a "non-verified purchase" means that the reviewer’s purchase is not confirmed. Since the latter category of reviews is not verified and in our model we considered them as ‘fake’. Hence the non-verified category review was detected as fake by our model as shown below in the Table 8 and 9.

**Table 8:** Indian Online Fashion Store Verified Customer Reviews (website link : https://www.reviews.io/company-reviews/store/myntra).

Sl No.	Review	Proposed Model’s Prediction	Inference Time
1	Myntra is the biggest scammer in Ecommerce world They fool customers mentioning easy return, Actually they do not really accept the return request, harass customers & at the end customer itself leaves to follow up. Have some shame Myntra… They deserve to be imprisoned for this kind of fraud	True	0.0218 s
2	They cancelled my order without informing me. The money was already paid. When I tried to login to my account they blocked me. And when i phoned them they said it was cancelled for “security” reasons. No other explanation given. They say they have initiated a process to refund my money by the 26 November. I’ll have to wait and see if they keep to their undertaking of the refund. The lady I spoke to by the name of Anjali was totally unhelpful. Please don’t deal with this company unless you want to be disappointed.	True	0.0240 s
3	Convinced fees charged … which is not right.	True	0.0203 s
4	Horrible experience. Pathetic customer service. Cheaters in the delivery chain. even the delivery person has been taught how to trick customers. we received a broken item. this is my last purchase from myntra. but the wayt hey are growing, they dont care.	True	0.0235 s
5	I am writing this post with deep disappointment and dissatisfaction regarding my recent experience with Myntra. Over a month ago, I returned two products, and yet, the company has not processed my refund. Despite calling customer support more than 10 times, each time I was assured a resolution within 48 h, but no action has been taken, and I received no further response. This is not the level of service one expects from a reputed platform like Myntra. It’s essential to hold companies accountable for their commitments to their customers.	Fake	0.0219 s

**Table 9:** Indian Online Fashion store  verified customer reviews (https://www.amazon.com/gp/help/customer/display.html?nodeId=G75XTB7MBMBTXP6W).

Sl No.	Review	Proposed Model’s Prediction	Inference Time
1	Beautiful simple shirt of fairly high quality made in Vietnam ( which I view as a positive) It is not light whatsoever. 100% cotton with a hand similar to a light flanel. Def. shrinks so size up .	True	0.0245 s
2	Sizing is a bit off, but for the price I would buy another. Cotton is pure and have a good thickness for its softness.	Fake	0.0234 s
3	I always find myself surprisingly pleased with the Amazon Essentials products. These shirts are extremely well made with substantial quality fabric and good color selections. A note about size: I’m a true medium but ordered large because I wanted a roomier fit. That’s exactly what I got. The neck and shoulders fit my medium frame perfectly but the body is cut a bit fuller and longer than a regular medium t-shirt would be… buuuutt, someone looking for a true to size large will likely find the shirt tight and too small.	Fake	0.0232 s
4	These are well made tees. Very nice material that washes well. True to size and doesn’t shrink. Gray heathered colored was as pictured. Would recommend.	True	0.0232 s
5	Every time I put this shirt on I tell myself I’m going to go back and write a review so other don’t make the same mistake. Pros: Very soft thick cotton. Warm and comfortable Should fit OK for someone a lot shorter than me.	True	0.0391 s

## Performance on Live e-commerce websites reviews

**Fig. 17:**
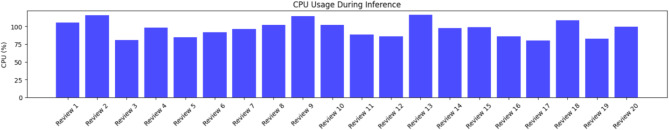
Memory Utilization during Inference.

**Fig. 18:**
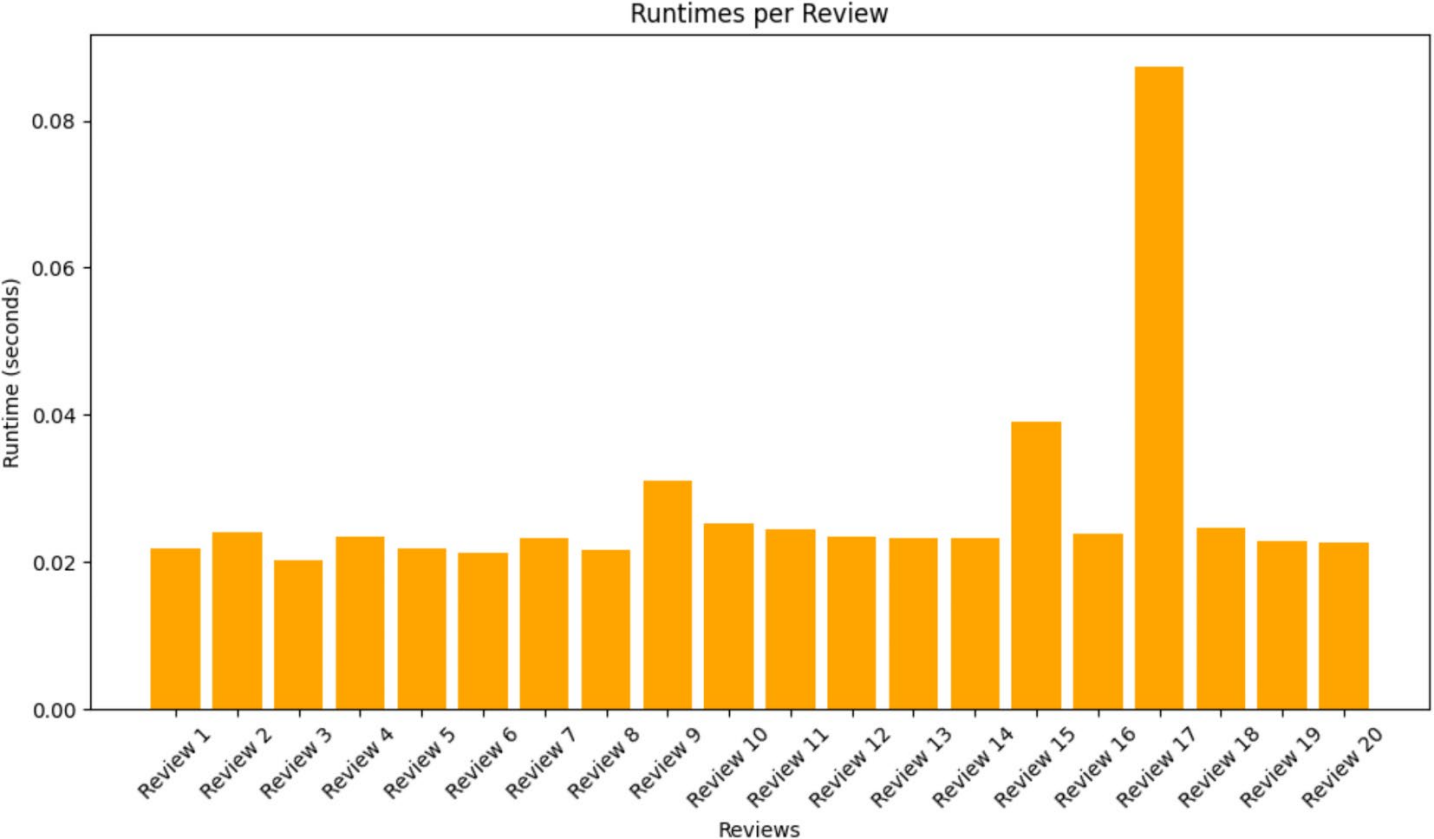
Runtime per review.

The above Table 10 shows the comparison of other existing models and latest transformer based models with the usage of same dataset and found that our proposed model MBO + DeBERTa excels in accuracy. proposed MBO + DeBERTa model was also tested on adversarial and noisy data with various combinations of 10, 20 and 50% attack rates over three review dataset and produced better performance in all metrics as shown in below Tables 11, 12 and 13. Figures 17 and 18 also shows the confusion matrix drawn against the normal and adversarial data. Thus the Robustness analysis were performed for the proposed model and achieved an effective results

The memory utilization and run time per reviews were also discussed in the below Figs. 17 and 18 as MBO and DeBERTa are computationally intensive.

**Table 10:** Comparative analysis of the same dataset with existing works.

Paper	Dataset used	Technique	Result (%)
Shojaee et al^44^.	Deceptive Opinion Dataset	Naive Bayes SAGE	84
Ahmed et al.^45^,	Deceptive Opinion Dataset	LSVM	90
**Proposed Model**	**Deceptive Opinion Dataset**	**MBO + DeBERTa**	**91**
Saumya, S. Singh, J. P^12^.	Amazon Dataset	LSTM Autoencoder	74
Loke et al.^46^,	Amazon Dataset	SVM	71
**Proposed Model**	**Amazon Dataset**	**MBO + DeBERTa**	**78**
Sujithra et al.^47^,	Deceptive Opinion Dataset	BERT	91
Cipirian et al.^48^,	Fake News Corpus	BART and RoBERTa	92.5

**Table 11:** Proposed model over the adverserial and noisy data.

Dataset	Normal data				Adversarial & Noisy Data			
	Accuracy	Precision	Recall	F1	Accuracy	Precision	Recall	F1
Amazon	78	77	78	77	77	77	78	77
Fake review	98	98	99	98	97	97	96	96
Deceptive Opinion	91	91	90	90	89	90	88	89

**Table 12:** Robustness analysis on noisy and erroneous data with 50% NOISE.

Sl No.	Original Review	Noisy and erroneous Review	Proposed Model’s Prediction on original review	Inference Time on Original review	Proposed Model’s Prediction on noisy and erroneous review	Inference Time on noisy and erroneous review
1	Myntra is the biggest scammer in Ecommerce world They fool customers mentioning easy return, Actually they do not really accept the return request, harass customers & at the end customer itself leaves to follow up.	Myntra is the rmunqkp scammer gi Ecommerce world xcoh fool customers muulavfcxv easy wvqdnnl rzbzonul they do pxx lkfmcw hnyace the return vkjwrvdm harass customers & at	True	0.0218 s	True	0.0237 s
2	They cancelled my order without informing me. The money was already paid. When I tried to login to my account they blocked me. And when i phoned them they said it was cancelled for “security” reasons. No other explanation given. They say they have initiated a process to refund my money by the 26 November. I’ll have to wait and see if they keep to their undertaking of the refund. The lady I spoke to by the name of Anjali was totally unhelpful. Please don’t deal with this company unless you want to be disappointed.	abiz cancelled my nbdec xnbbhvy informing isw uhb fyssv was already vrxtc When I wgyho to login to al olnopyg keao ajoltaz me. And ruhs x howqrj them byxy said it ssz awqotahpd for ‘security’ reasons. tg ipzcf explanation given. They say hztz have nwimotgqe a iuukjuu sp satrky my money db vpm 26 November. I’ll have to eynq kbk see if qvhb fchj to ruezj sntpyyajkdh zt jky smxlfiv akm lady t spoke to rx the goks of Anjali wgz tfbvihc unhelpful. Please don’t iiit with this cqhqrqa dnfhdb fgq want lq be disappointed.	True	0.0240 s	True	0.0232 s
3	Convinced fees charged … which is not right.	Convinced fofi charged … nsmfd go not xjcdvh.	True	0.0203 s	True	0.0201 s
4	Horrible experience. Pathetic customer service. Cheaters in the delivery chain. even the delivery person has been taught how to trick customers. we received a broken item. this is my last purchase from myntra. but the wayt hey are growing, they dont care.	txlclnfr experience. Pathetic customer nvpzqosx zjamiurj in the delivery ffocto even the delivery cfbowh msl been lvjgil how to cjedq customers. ft received h hsjqzm item. nxez ij dg last purchase from myntra. but bsr hzek rgs gqf growing, snxd vnuy qvrnz	True	0.0235 s	True	0.0228 s
5	I am writing this post with deep disappointment and dissatisfaction regarding my recent experience with Myntra. Over a month ago, I returned two products, and yet, the company has not processed my refund. Despite calling customer support more than 10 times, each time I was assured a resolution within 48 h, but no action has been taken, and I received no further response. This is not the level of service one expects from a reputed platform like Myntra. It’s essential to hold companies accountable for their commitments to their customers.	I bv writing this rckg with tcbz dojqcvwedxpfgo yyr dissatisfaction regarding my yuxntu zgtetpmvah with wpuxhti Over s month ago, z returned oyx qgxcxfqrj and yet, the ftdpwhz has not processed my refund. qstnbce gebmczs drbsbkso mlpxoha more uerj mu times, each time I was chytxic b resolution nzixgf kp hours, but cl action has been taken, and y received no vgbhtdr medepoqxd This gt not cjv level vr service thi expects from u fwgstsj lbclnnno like sakucho It’s fcgefzmet ed fujm ymecztzgv xdbkeetjvyi qug hjehn jpxrqurxqcg ox their vkjzbkzfur	Fake	0.0219 s	Fake	0.0230 s

**Table 13:** Robustness analysis to adversarial attacks:.

Sl No.	Original Review	Adversarial Review	Proposed Model’s Prediction on original review	Inference Time on Original review	Proposed Model’s Prediction on adversarial Review	Inference Time on adversarial Review
1	Myntra is the biggest scammer in Ecommerce world They fool customers mentioning easy return, Actually they do not really accept the return request, harass customers & at the end customer itself leaves to follow up. Have some shame Myntra… They deserve to be imprisoned for this kind of fraud	Myntra is **such** a gigantic **deceiver** in the E-commerce field. They “mention” **simple return policies** but NEVER accept return requests. Customers are harassd continously until they gv up followups. No respect Myntra, have **sum shame…** Jail-time is the **LEAST** this fraud deserves.	True	0.0218 s	True	0.0210 s
2	They cancelled my order without informing me. The money was already paid. When I tried to login to my account they blocked me. And when i phoned them they said it was cancelled for “security” reasons. No other explanation given. They say they have initiated a process to refund my money by the 26 November. I’ll have to wait and see if they keep to their undertaking of the refund. The lady I spoke to by the name of Anjali was totally unhelpful. Please don’t deal with this company unless you want to be disappointed.	They cancelled my order without **notice! Prepaid cash gone.** Tried logging in? Accounnt BLOCKED. On call, they mentioned vague “security” issues. No **clarity** provided. Now they **promise refund** by 26th Nov; let’s SEE if they mean it. The rep I spoke to, Anjalli, was unhlpful nd rude. Avoid this co at all costs **or get heartbroken**.	True	0.0240 s	True	0.0268 seconds
3	Convinced fees charged … which is not right.	Extra fees dedcted! Totally NOT justified	True	0.0203 s	True	0.0214 seconds
4	Horrible experience. Pathetic customer service. Cheaters in the delivery chain. even the delivery person has been taught how to trick customers. we received a broken item. this is my last purchase from myntra. but the wayt hey are growing, they dont care.	Terrible exp. Poor custmr care! Delivery ppl **literally TRAINED to** scam us. Item? BROKEN. Myntra’s greed GROWSS—last buy frm here! buttt, **do they even care? No.**	True	0.0235 s	True	0.0233 s
5	I am writing this post with deep disappointment and dissatisfaction regarding my recent experience with Myntra. Over a month ago, I returned two products, and yet, the company has not processed my refund. Despite calling customer support more than 10 times, each time I was assured a resolution within 48 h, but no action has been taken, and I received no further response.	Deeply disappointed!!! Returned TWO products **30 + days ago**, still no REFUND. Called cust.support **10x**! Everytime: “48 hrs we’ll fix!” ZERO action. No follow-ups. Totally unacceptable for a platform as “reputable” as Myntra. Companies **MUST** be held accountable **or else customers suffer.**	Fake	0.0219 s	Fake	0.0226 s
6	Myntra is running some scam. their pick up agents are used to run these scams. I will highly recommend not to buy. fake sellers and then their fake products are being not returned	Myntra’s operations feel highly **questionable.** Their so-called ‘pick-up agents’ seem to have some **unprofessional** practices, making returns impossible. I STRONGLY RECOMMEND avoiding this platform altogether. It appears to prioritize sellers with **questionable authenticity**, pushing subpar products that conveniently can’t be returned. A highly disappointing experience.	True	0.0212 s	True	0.0218 s

## Conclusion and future work

The MBO-DeBERTa classification model proves to be a sophisticated approach to improve the Fake review detection accuracy. The integration of a novel positional data describing the review text structure from DeBERTa and enhancing the new solution’s fitness using MBO, the proposed model MBO-DeBERTa improves its ability to accurately classify fake reviews. This proposed model enhances the capability to differentiate between overlapping features of fake and genuine reviews. MBO-DeBERTa achieved a classification accuracy of 98% for detecting the fake reviews. Thus the suggested model obtained 98% accuracy, 98% precision, 97% recall ,97% F1 score and low loss rate of 0.22%.when compared to the previous models. Overall, this comprehensive approach holds promise for advancing the detection of fake reviews. The proposed model also detected adversarial attacks using the Fast Gradient Sign Method (FGSM) and thereby evaluating its resistance to such attacks and noise. The proposed model was also tested on the unseen data of Myntra and Amazon verified customer reviews and our model works efficiently for real world data. Thus the results show that the suggested model outperforms the current models showing increased accuracy, precision, recall, F1 score and reduced loss rate. A promising future work direction would be to investigate methods for fusing multi-modal data sources, beyond review features such as demographic attributes and ratings to improve the accuracy and reliability of fake review detection.

## Data Availability

Data is provided within the manuscript. Data was taken from the below links and citation has been provided in the reference section of the manuscript1.https://www.kaggle.com/datasets/rtatman/deceptive-opinion-spam-corpus2.https://www.kaggle.com/datasets/naveedhn/amazon-product-review-spam-and-non-spam3.https://www.kaggle.com/datasets/mexwell/fake-reviews-dataset.
